# Development and validation of a multimodal interpretable machine learning model for the identification of osteoporosis in patients with type 2 diabetes mellitus: a multicenter retrospective study

**DOI:** 10.3389/fendo.2026.1871923

**Published:** 2026-07-01

**Authors:** Jihao Cheng, Chuanjiang Liu, Mengyin Gu, Dongying Su, Jingyun Liao

**Affiliations:** 1 Department of Radiology, The Second Affiliated Hospital of Zhejiang Chinese Medical University, Hangzhou, China; 2 Department of Endocrinology, the Second Affiliated Hospital of Zhejiang Chinese Medical University, Hangzhou, China; 3 Department of Radiology, Affiliated Xiaoshan Hospital, Hangzhou Normal University, Hangzhou, China

**Keywords:** machine learning, multicenter study, osteoporosis, SHAP, type 2 diabetes mellitus

## Abstract

**Background:**

Osteoporosis is a prevalent yet underdiagnosed complication in patients with type 2 diabetes mellitus (T2DM), significantly increasing the risk of fractures and mortality. However, current screening tools are limited by low accuracy and lack of generalizability. This multicenter cross-sectional study aimed to develop and validate a multimodal interpretable machine learning (ML) model integrating multimodal data to identify and classify osteoporosis risk in T2DM patients, with a focus on clinical translatability.

**Methods:**

We retrospectively enrolled 1,002 T2DM patients from two tertiary hospitals in China. After exclusions, Cohort 1 (n = 852) was used for model development and internal validation, and Cohort 2 (n = 126) served as an independent external validation set. Multimodal data included demographics, laboratory tests, abdominal CT-derived parameters (e.g., skeletal muscle index at L3, SMI-L3), and composite metabolic indices (e.g., lymphocyte-to-HDL ratio [LHR], Metabolic Score for Visceral Fat [METS-VF]). Core predictors were selected using univariate analysis, Least Absolute Shrinkage and Selection Operator (LASSO) regression, and the Boruta algorithm. Seven ML algorithms were compared, with model performance evaluated by area under the receiver operating characteristic curve (AUC), accuracy, sensitivity, specificity, F1-score, and area under the precision-recall curve (AUPRC). The SHapley Additive exPlanations (SHAP) method was applied for model interpretability.

**Results:**

Seven predictors were identified: age, hemoglobin, neutrophil count, uric acid, LHR, SMI-L3, and METS-VF. The eXtreme Gradient Boosting (XGBoost) model demonstrated superior performance, achieving an AUC of 0.877 (95% confidence interval [CI]: 0.830–0.923) in the training set and 0.911 (95% CI: 0.879–0.943) in the external validation set. SHAP analysis revealed age, hemoglobin, and uric acid as the top contributors. Calibration and decision curve analyses confirmed the model’s clinical utility.

**Conclusion:**

This study presents a robust, multimodal interpretable ML model for osteoporosis risk prediction in T2DM using routinely available clinical data. By integrating multimodal features and providing transparent predictions via SHAP, the model offers a proof-of-concept tool for opportunistic screening to facilitate targeted dual-energy X-ray absorptiometry (DXA) referral, although prospective clinical validation is needed before its clinical utility can be established.

## Introduction

Diabetes mellitus remains a major global public health challenge, currently affecting approximately 537 million people worldwide. In China, the prevalence of diabetes has reached 12.8%, affecting an estimated 150 million individuals, with type 2 diabetes mellitus (T2DM) accounting for over 90% of cases (1, 2).

Hormonal imbalances and metabolic disturbances in T2DM patients contribute to various complications. Specifically, insulin resistance, accumulation of advanced glycation end products (AGEs), oxidative stress, and chronic inflammation collectively impair bone metabolism, leading to decreased bone formation and increased bone resorption—ultimately resulting in osteoporosis. Osteoporosis is a metabolic bone disorder characterized by trabecular degeneration, microarchitectural deterioration, reduced bone mass, and increased bone fragility, and is one of the most common complications of T2DM (3). Persistent hyperglycemia accelerates calcium loss, disrupts bone metabolism, and contributes to diabetic osteoporosis. Accumulating evidence supports an association between osteoporosis and metabolic diseases such as T2DM, hypertension, and hyperlipidemia (4). Individuals with these conditions often exhibit systemic inflammation, oxidative stress, insulin resistance, and endothelial dysfunction, all of which can impair bone remodeling and reduce bone mineral density (5). Studies by Pahk et al. have shown that organ fat accumulation is linked to subclinical chronic inflammation, and the adipokines produced appear to influence bone metabolism (6). Furthermore, pro-inflammatory cytokines such as interleukin-1 (IL-1), interleukin-6 (IL-6), and tumor necrosis factor-alpha (TNF-α) are generated, triggering systemic inflammatory responses that negatively impact bone metabolism (7, 8).

Diabetic osteoporosis significantly elevates the risk of falls and fractures, thereby increasing the likelihood of disability, hospitalization, and mortality (9, 10). In China, the estimated prevalence of osteoporosis among T2DM patients is 35%, and this figure is projected to rise to 40.8% by 2030, posing a pressing public health issue (11, 12). Although bone mineral density screening tools such as dual-energy X-ray absorptiometry (DXA) are accurate, their high cost and limited accessibility hinder large-scale application. Therefore, developing an economical, convenient, and easily implementable early screening tool for osteoporosis—particularly for primary care settings—is of paramount importance for T2DM patients.

Currently, there is a lack of osteoporosis risk prediction tools that integrate multimodal clinical data, offer robust generalizability, and provide clinical interpretability. Advances in artificial intelligence (AI), particularly machine learning (ML), offer novel approaches for analyzing complex, multidimensional medical data (13, 14). Compared to traditional statistical methods, ML algorithms excel at integrating high-dimensional clinical features, capturing nonlinear relationships, and modeling interactions among variables, thereby enabling the construction of more accurate and robust predictive models (15). However, most existing studies are limited by single-center designs, small sample sizes, and lack of external validation, compromising model generalizability. Moreover, these models often function as “black boxes” with limited interpretability, restricting their clinical adoption. Incorporating independent external validation datasets and explainable AI techniques, such as SHapley Additive exPlanations (SHAP), can enhance model transparency and elucidate the contribution of individual features to predictions (16, 17). To address these gaps, this study integrates multimodal clinical data—including demographics, laboratory tests, imaging parameters, and derived metabolic indices—using multi-step feature selection and multiple machine learning algorithms to construct and externally validate a multimodal interpretable osteoporosis risk prediction model. Given the cross-sectional nature of the data, this model is designed to serve as an efficient, transparent, and practical screening adjunct to support the identification of existing osteoporosis based on routinely available clinical profiles, thereby facilitating targeted screening and intervention.

## Methods

### Study population

#### Study design and participants

This multicenter retrospective study enrolled 1,002 patients with T2DM from two tertiary hospitals in China. Cohort 1 (derivation cohort) consisted of 852 patients from the Second Affiliated Hospital of Zhejiang Chinese Medical University (January 2023 to August 2025). This cohort was randomly stratified by osteoporosis status and split into a training set (n = 682, 80%) and an internal validation set (n = 170, 20%). Cohort 2 (external validation cohort) comprised 126 independent patients from Zhejiang Xiaoshan Hospital (January 2024 to August 2025), which was used solely to assess the generalizability of the final model.

Inclusion criteria were (1) age ≥ 45 years; (2) diagnosis of T2DM according to WHO criteria (18); (3) completion of standardized DXA assessment; (4) availability of complete electronic medical records during hospitalization, including demographic data and routine laboratory results; (5) the full abdominal CT scan was performed during hospitalization. Exclusion criteria were: (1) secondary osteoporosis; (2) hematological system diseases; (3) history of malignant tumors; (4) severe hepatic or renal insufficiency; (5) acute infectious diseases; (6) incomplete clinical data; (7) use of medications such as hormone replacement therapy, bisphosphonates, glucocorticoids, or proton pump inhibitors.

This study was conducted in accordance with the Declaration of Helsinki and the Strengthening the Reporting of Observational Studies in Epidemiology (STROBE) guidelines for observational studies. The retrospective analysis was approved by the ethics committees of both institutions (Ethics approval numbers: Cohort 1: 2026-014-01-K; Cohort 2: KL-2026-002-01). The requirement for written informed consent was waived due to the de-identified and retrospective nature of the analysis using anonymized data.

#### Sample size estimation

To ensure robust model development and minimize overfitting, sample size was calculated using the Riley framework via the *pmsampsize* package in R. Based on prior evidence that the incremental predictive value of new models for osteoporosis risk in T2DM patients beyond traditional factors is typically low, we conservatively set the anticipated Cox-Snell R² at 0.015. This value was informed by prior T2DM osteoporosis prediction models that reported low incremental explained variance beyond traditional risk factors (e.g., Cox-Snell R² ≈ 0.015) (2). Furthermore, Riley et al. have recommended that when precise prior estimates of model performance are unavailable, a conservative (i.e., low) R² should be assumed to ensure an adequately powered sample size, as overestimating R² risks underpowering the study (2, 19).

With a conservative estimate of 10 candidate predictors anticipated to be included in the final model and an anticipated osteoporosis prevalence of approximately 35% in T2DM patients derived from the literature, the calculated required sample size was approximately 650 participants. Accounting for a potential 20% rate of missing data, the target sample size for the model training cohort was set at no less than 780 patients. Cohort 1 in this study included 852 T2DM patients, exceeding the calculated requirement and satisfying the sample size needed for model development. While R² = 0.015 is a highly conservative assumption, this deliberate approach aligns with Riley et al., who caution that underestimating R² yields a safer sample size whereas overestimating risks underpowering. This conservatism is retrospectively justified: the final model achieved AUCs of 0.877 (training) and 0.911 (external validation), far exceeding the performance implied by R² = 0.015; moreover, the EPV ratio of approximately 49:1 (344 events/7 predictors) substantially surpasses the recommended minimum of 10–20, confirming adequate sample size regardless of the initial R² assumption.

### Data collection and definitions

#### Demographics and laboratory tests

Baseline demographic characteristics including age, sex, height, weight, and waist circumference were recorded. Complete blood counts, including hemoglobin (HGB), neutrophil count (NEUT), red blood cell count (RBC), platelet count (PLT), lymphocyte count (LYMPH), and monocyte count (MONO), were measured using a Mindray BC-6800Plus hematology analyzer. Blood biochemical parameters were determined using an automated biochemical analyzer (Beckman Coulter AU5800, Brea, CA, USA), encompassing total cholesterol (TC), triglycerides (TG), high-density lipoprotein cholesterol (HDL-C), low-density lipoprotein cholesterol (LDL-C), alanine aminotransferase (ALT), aspartate aminotransferase (AST), gamma-glutamyl transferase (GGT), lactate dehydrogenase (LDH), alkaline phosphatase (ALP), uric acid (UA), fasting plasma glucose (FPG), serum potassium (K), serum sodium (Na), serum calcium (Ca), serum phosphorus (Pi), serum magnesium (Mg), creatinine (Cr), postprandial blood glucose (PBG), fasting insulin (FINS), fasting C-peptide (FCP), and glycated hemoglobin (HbA1c). Four tumor markers, AFP, Ca125, Ca199, and CEA, were measured using a Roche cobas 8000 e602 electrochemiluminescence immunoassay analyzer. All laboratory results were extracted from the initial tests performed within 24 hours of hospitalization.

#### Osteoporosis assessment

Bone mineral density (BMD) at the lumbar spine (L1-L4), left femoral neck, and total hip was assessed using DXA (Hologic, Discovery, WI, USA). All DXA scans were performed by trained and certified technicians. Participants were classified into the osteoporosis group (T-score ≤ -2.5) and the non-osteoporosis group (T-score > -2.5) according to the World Health Organization (WHO) criteria based on T-scores (20).

#### CT imaging parameters and body composition analysis

All CT examinations were performed using a Siemens Somatom Perspective 64-slice spiral CT scanner. The acquisition parameters for non-contrast abdominal CT scans were: detector configuration 0.625 mm × 64, 120 kV, 300 mA, slice thickness 1.0 mm, slice interval 1.0 mm, and pitch 0.984. The field of view covered the abdomen, extending from the xiphoid process to the pubic symphysis.

Hepatic steatosis (FLD) was assessed on non-contrast CT images by measuring liver density. Regions of interest (ROIs) were placed on slices with the largest possible homogeneous liver parenchyma, avoiding hepatic vessels and artifacts. Three measurements were taken (one in the left lobe, two in the right lobe), and the mean value was calculated. Hepatic steatosis was defined as a mean liver attenuation value < 48 Hounsfield units, consistent with previous reports (21).

Intrapancreatic fat deposition (IPFD) was evaluated by measuring CT attenuation values of the pancreas and spleen on CT images (see Supplementary Material, **Supplementary Figure 1**). An ROI of approximately 0.5 cm² was placed on the pancreatic body and tail at the level of the mid-T12 vertebral body. The mean pancreatic CT attenuation was recorded. Splenic CT attenuation was measured at the same anatomical level, and the pancreas-to-spleen attenuation ratio (P/S ratio) was calculated. According to the study by Kim et al., pancreatic steatosis was defined as a P/S ratio < 0.7 (22, 23), as detailed in **Supplementary Figure 1**.

Fully automated segmentation of axial CT images at the third lumbar vertebra (L3) level was conducted using AI-based abdominal tissue segmentation software (uAI Discover PLBMD; United Imaging Intelligence, Shanghai, China), as illustrated in **Supplementary Figure 2**. This software, based on the VB-Net algorithm and employing threshold-based segmentation, automatically and accurately delineated the visceral fat area (VFA), subcutaneous fat area (SFA), and paraspinal muscle cross-sectional area (CSA). The skeletal muscle index (SMI) was calculated using the CSA: SMI = CSA (cm²)/height² (m²).

Notably, the full abdominal CT scans analyzed in this study were not performed for research purposes. Given the retrospective design, all CT images were obtained from existing clinical examinations conducted as part of the patients’ routine inpatient care prior to or during the index hospitalization. The primary clinical indications for these CT scans included, but were not limited to, the evaluation of non-specific abdominal symptoms, screening for diabetic complications (e.g., assessment for hepatic steatosis or renal calculi), pre-operative assessment, or further investigation of incidental findings. This study retrospectively leveraged these existing images for secondary analysis of body composition, including the derivation of SMI-L3 and assessment of intra-pancreatic fat deposition. Consequently, this approach reflects the opportunistic use of real-world clinical data rather than a prescribed screening protocol for all T2DM patients.

### Derivation of composite metabolic indices

To capture interactions related to obesity and metabolic-inflammatory status, six composite indices and the Metabolic Score for Visceral Fat (METS-VF) were derived from routine laboratory measurements. The six composite indices were: monocyte-to-HDL ratio (MHR), neutrophil-to-HDL ratio (NHR), platelet-to-HDL ratio (PHR), lymphocyte-to-HDL ratio (LHR), triglyceride-glucose index (TyG), and cholesterol-glucose index (CHG). The derivation formulas for these six indices, based on methodologies described in the literature (24–30), are as follows:

MHR = MONO(×10^9/^L)/HDL-C(mmol/L)
NHR = NEUT(×10^9/^L)/HDL-C(mmol/L)
ΛPHR = PLT(×10^9/^L)/HDL-C(mmol/L)
LHR = LYMPH(×10^9/^L)/HDL-C(mmol/L)
TyG = ln [TG (mg/dL) × FPG (mg/dL)/2]
CHG = ln [TC (mg/dL) × FPG (mg/dL)/(2 × HDL-C (mg/dL))]

The Metabolic Score for Visceral Fat (METS-VF) is a novel surrogate marker for evaluating visceral and abdominal adiposity. It integrates fasting plasma glucose (FPG), triglycerides (TG), high-density lipoprotein cholesterol (HDL-C), body mass index (BMI), height, waist circumference (Waist), sex, and chronological age (31). The specific calculation formula is as follows:

METS-VF = 4.466 + 0.011 × [ln(METS-IR)]³ + 3.239 × [ln(WHtR)]³ + 0.319 × Sex + 0.594 × ln(age).

^1.^METS-IR = Ln[(2 × FPG + TG) × BMI/Ln(HDL-C)], with all biochemical values in mg/dL.
^2.^WHtR = WC/HT, referred to as Waist-to-Height Ratio, defined as waist circumference (WC, cm) divided by height (cm).
^3.^Sex is coded as 1 for males and 0 for females

### Data preprocessing

A three-tier data quality control protocol was implemented (1): variables with >20% missing values were excluded; (2) For continuous variables, outliers were initially identified using the 1.5 times interquartile range (IQR) rule, followed by Winsorization at the 1st and 99th percentiles for all continuous variables to mitigate the impact of extreme values on model training. (3) Remaining missing values were imputed using the Random Forest algorithm (10 iterations, tree depth = 100). Prior to imputation, the overall missing data proportion in Cohort 1 was low. Specifically, 58 of the 67 collected variables (86.6%) exhibited a missing rate below 5%. The only variable with a missing rate exceeding 10% was postprandial blood glucose (PBG, 15.2%), while CA125 and CA199 showed missing rates of 12.3% and 11.8%, respectively. Variables with >20% missing data were excluded from the analysis per the prespecified protocol. Remaining missing values were imputed using the Random Forest algorithm (10 iterations, tree depth = 100). To assess the validity of the imputation, density plots were used to compare the distribution consistency of variables before and after imputation, and imputation errors were evaluated based on cross-validation to ensure the imputed data possessed good representativeness and stability. Following imputation, all continuous variables were standardized using Z-Score normalization based on the mean and standard deviation calculated from the training set. Categorical variables were converted into dummy variables via one-hot encoding.

Regarding class imbalance, the distribution of the outcome variable in the training set was evaluated prior to model development. The osteoporosis group (n = 344) and non-osteoporosis group (n = 338) were nearly balanced (proportion of the minority class = 49.4%), with an imbalance ratio of approximately 1:1.02. Given the absence of substantial class imbalance (commonly defined as a minority class proportion < 40%), no resampling techniques (e.g., SMOTE, random oversampling, or undersampling) or class-weight adjustments were applied. This decision was further supported by the stratified splitting procedure, which preserved the osteoporosis prevalence across the training and internal test sets.

### Feature selection

**Figure 1:**
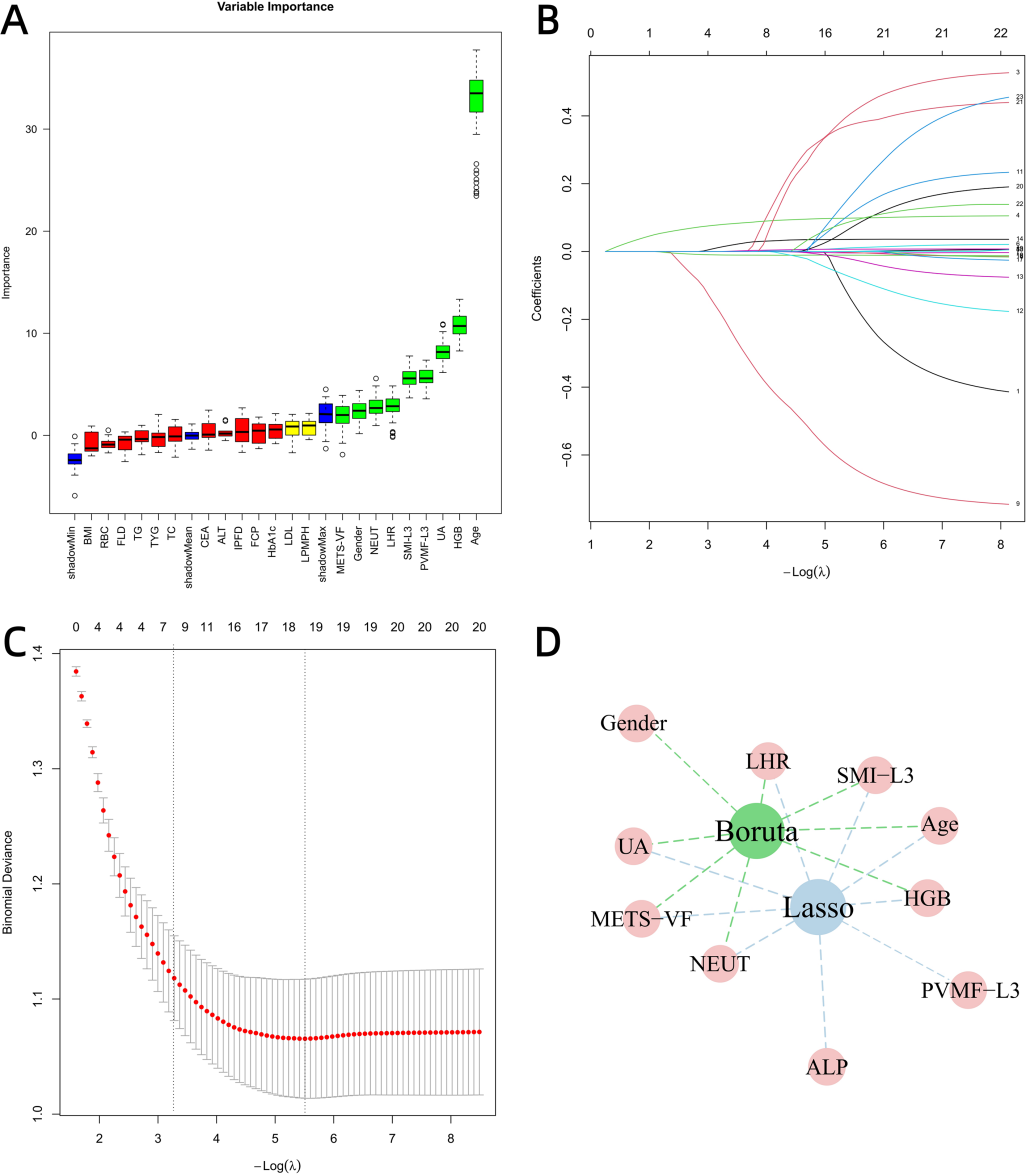
Feature selection with Boruta and LASSO regression. **(A)** The blue plot shows minimum, average, and max shadow score. Variables having box plot in green are important, in yellow as tentative, and in red as rejected; **(B)** The correlation between L1 norm and different coefficients in LASSO regression. L1 norm is the regularisation term for LASSO; **(C)**. The correlation between lambda with binomial deviance. There are two dashed lines in the graph. The left dashed line indicates the minimum mean squared error while the right one indicates one standard error away from the minimum mean squared error; **(D)**. The network Venn diagram illustrates the overlapping predictors identified by LASSO and Boruta screening.HGB, hemoglobin; BMI, body mass index; RBC, red blood cell count; LYMPH, lymphocyte count; NEUT, neutrophil count; TC, total cholesterol; TG, triglycerides; LDL-C, low-density lipoprotein cholesterol; ALT, alanine aminotransferase; HbA1c, haemoglobin A1c; UA, uric acid; FCP, fasting c peptide; LHR, Lymphocyte-to-HDL Ratio; TyG, Triglyceride-Glucose Index; CEA, carcinoembryonic antigen; FLD, fatty liver disease; IPFD, intra-pancreatic fat deposition; METS-VF, metabolic score for visceral fat; SMI-L3, skeletal muscle index of the third lumbar vertebra; CSA-L3 cross sectional area of the third lumbar vertebra.

Cohort 1, used for model development, was randomly stratified by osteoporosis status into a training set (80%) and an internal test set (20%). Cohort 2 served as the external validation set. First, univariate logistic regression was performed on the training set; variables with P < 0.05 were retained for further selection. Subsequently, the Boruta algorithm and Least Absolute Shrinkage and Selection Operator (LASSO) regression were applied in parallel to these candidate variables for feature selection. The Boruta algorithm, a wrapper built around a Random Forest (RF) classifier, was implemented using the “Boruta” package to iteratively assess variable importance and retain relevant features (32). LASSO regression was conducted using the “glmnet” package in R (version 4.1.10). Given the binary nature of the outcome variable, a binomial logistic LASSO regression model (family = “binomial”) was employed. To determine the optimal regularization parameter λ, ten-fold cross-validation was performed within the training set. The λ value that minimized the binomial deviance (i.e., the minimum cross-validation error, λ.min) was selected as the final parameter to ensure model predictive accuracy (33). Variance inflation factor (VIF) scores were calculated, and variables exhibiting high collinearity (VIF > 10) were removed to maintain model stability. The LASSO regression coefficients and VIF values for all candidate variables are provided in **Supplementary Table 2**.The intersection of features selected by both LASSO and Boruta was considered as robust core predictors (**Figure 1D**). These were then considered alongside clinical relevance, culminating in the selection of seven final features for subsequent machine learning model development.

### Model development and evaluation

Seven machine learning algorithms were employed to construct predictive models: eXtreme Gradient Boosting (XGBoost), Logistic Regression (LR), Light Gradient Boosting Machine (LightGBM), Gradient Boosting Decision Tree (GBDT), Multilayer Perceptron (MLP), Gaussian Naive Bayes (GNB), and Support Vector Machine (SVM). Hyperparameters were optimized via grid search, with manual adjustments applied to learning rate, tree depth, and other regularization parameters. The optimized hyperparameters for each model are detailed in **Supplementary Table 3**. Taking XGBoost as an example, the grid search range included learning rate (0.01, 0.05, 0.1), maximum depth (3, 5, 7), subsample ratio (0.6, 0.8, 1.0), column sampling ratio (0.6, 0.8, 1.0), and number of trees (50, 100, 200). The hyperparameter search ranges for the other models are also listed in the **Supplementary Material**.

The performance of the optimal model was assessed using multiple metrics: Receiver Operating Characteristic (ROC) curve, accuracy, precision, sensitivity, specificity, F1 score, and the area under the Precision-Recall curve (AUPRC). Model calibration was assessed using calibration plots (reliability curves), which compare predicted probabilities against observed frequencies. A well-calibrated model was defined as one in which the calibration curve closely approximated the 45-degree diagonal reference line (representing perfect agreement between predicted and observed probabilities), consistent with the recommendations of the TRIPOD guidelines for prediction model reporting (19).

To obtain an unbiased estimate of the optimal model’s performance and mitigate overfitting, 10-fold nested cross-validation was implemented to enhance model generalizability. The training set was randomly partitioned into ten equal subsets; in each iteration, nine subsets were used for training and one for validation. Prior to formal training, 15% of the overall data was randomly withheld as an independent test set. Within the nested cross-validation framework, the inner loop was responsible for selecting the optimal combination of hyperparameters using the training and validation sets, while the outer loop was used for final performance evaluation on the held-out test set.

### Model interpretation

This study quantified the contribution and importance of each variable to the final classification outcome by analyzing Shapley Additive exPlanations (SHAP) values computed on the training set. Computing SHAP on the training set reflects the feature importance patterns learned by the model during development, and is consistent with the standard practice for interpreting model behavior (34, 35). A higher SHAP value indicates greater importance of the corresponding feature for the model’s prediction output. This method includes SHAP summary plots (displaying variable importance) and SHAP dependence plots (exploring interactions between variables). These plots visually illustrate how the levels of each variable and their SHAP values interact with other predictors to elucidate the predictions generated by the optimal model. To further validate the generalizability of the identified feature importance, SHAP values were also computed on the external validation set, and the resulting feature rankings were compared for consistency.

### Statistical analysis

All statistical analyses were conducted using R (version 4.2.3) and Python (version 3.11.4). Continuous variables with a normal distribution were presented as mean ± standard deviation and compared using the independent samples t-test. Non-normally distributed variables were presented as median (interquartile range) and compared using the Wilcoxon rank-sum test. Categorical variables were expressed as frequencies and percentages and were compared using the Chi-square test or Fisher’s exact test, as appropriate. During the univariate analysis phase, to mitigate the risk of false positives potentially arising from multiple comparisons, a P-value < 0.05 was adopted as the preliminary screening threshold. Subsequent multi-step feature selection using LASSO regression and the Boruta algorithm further eliminated irrelevant variables, ensuring the robustness of the final selected predictors.

## Results

### Baseline characteristics

**Figure 2:**
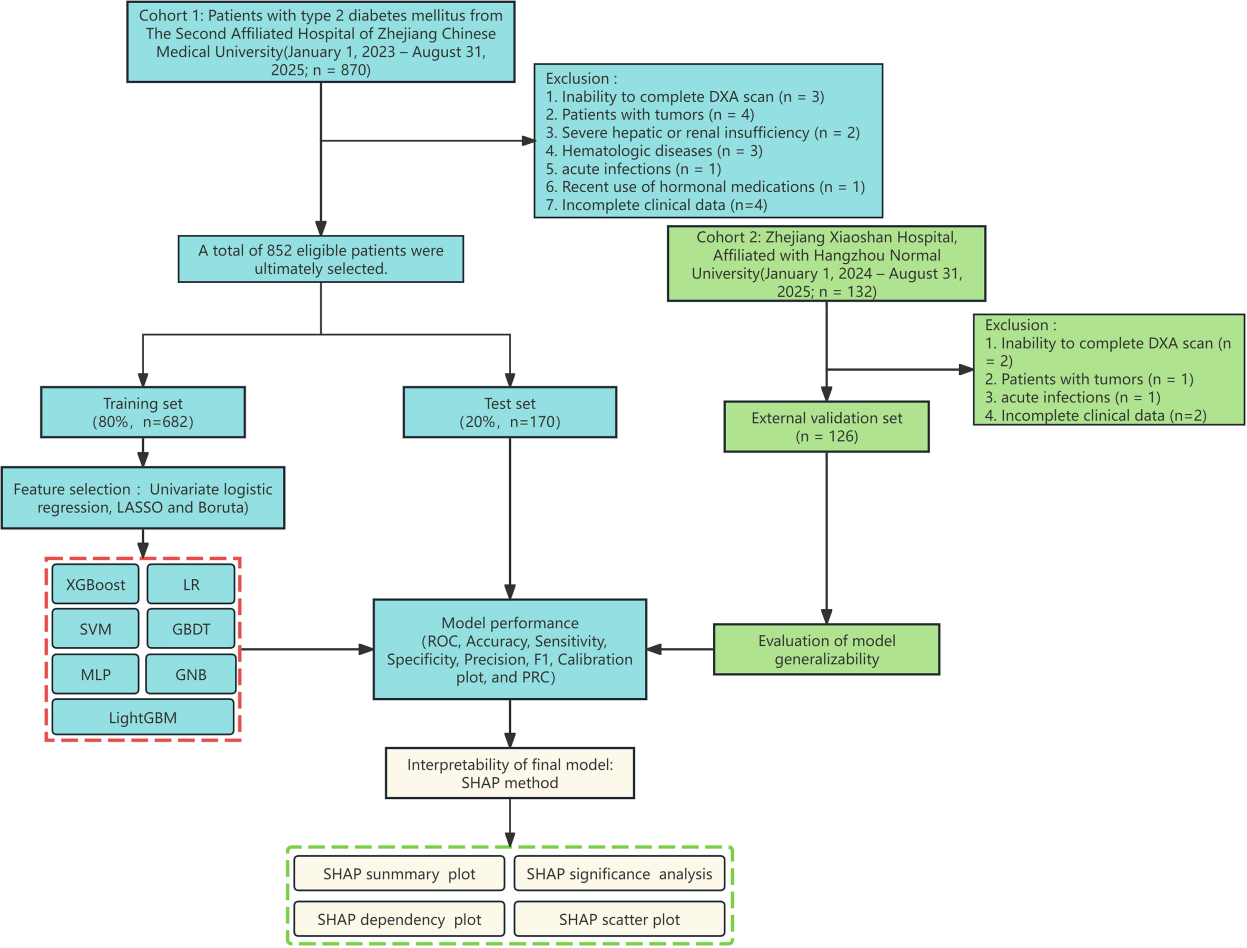
Study flow chart. XGBoost, extreme gradient boosting; LR, logistic regression; LightGBM, lightweight gradient boosting machine, GBDT, gradient boosting decision tree; MLP, multi-layer perceptron; GNB, Gaussian Naive Bayes; SVM, support vector machine; ROC, receiver operating characteristic curves; PRC, Precision Recall Curve; DCA, decision curve analysis; SHAP, Shapley additive explanations.

As shown in **Figure 2**, a total of 852 patients with T2DM from Cohort 1 were enrolled and stratified by bone mineral density (BMD) into an osteoporosis group (n = 424, T-score ≤ -2.5) and a non-osteoporosis group (n = 428, T-score > -2.5). Cohort 1 was subsequently randomly stratified by osteoporosis status into a training set (n = 682) and an internal test set (n = 170) in an 8:2 ratio. Baseline characteristics were well-balanced between the training and test sets, with no statistically significant differences observed (*P* > 0.05 for all variables; **Table 1**). Cohort 2, the independent external validation set, comprised 126 patients (osteoporosis group, n = 64; non-osteoporosis group, n = 62), with their characteristics detailed in **Supplementary Table 1**.

**Table 1:** Baseline characteristics of patients.

Variables	Total (n = 852)	Train (n = 682)	Test (n = 170)	P
Gender(%)				0.566
Male	530 (62.21)	420 (61.58)	110 (64.71)	
Female	322 (37.79)	262 (38.42)	60 (35.29)	
IPFD(%)				0.129
Yes	618(72.54)	482 (70.67)	136 (80.00)	
No	234 (27.46)	200 (29.33)	34 (20.00)	
FLD(%)				0.785
Yes	288 (33.80)	232 (34.02)	56 (32.94)	
No	564 (66.20)	450 (65.98)	114 (67.06)	
Age	64.00 (48.25, 68.00)	65.27 (50.75, 71.32)	64.13 (49.50, 69.75)	0.696
BMI(kg/m2)	24.60 (22.49, 27.30)	24.60 (22.50, 27.33)	24.40 (22.40, 27.08)	0.547
SFA-L3(cm2)	90.95 (64.23, 132.88)	89.85 (64.90, 130.85)	92.15 (63.00, 134.50)	0.786
VFA-L3(cm2)	121.35 (84.90, 164.90)	120.65 (84.85, 164.70)	129.20 (84.95, 164.97)	0.536
CSA-L3(cm2)	41.25 (34.82, 51.10)	41.30 (35.27, 50.73)	41.10 (33.85, 55.33)	0.939
SMI -L3(cm2/m2)	34.81 (30.36, 40.85)	34.75 (30.51, 40.49)	35.23 (29.43, 42.49)	0.689
RBC(×10^9^/L)	4.49 ± 0.64	4.45 ± 0.64	4.54 ± 0.60	0.113
HGB(×10^9^/L)	137.00 (123.25, 150.00)	136.00 (122.00, 149.00)	142.50 (127.25, 151.75)	0.061
PLT(×10^9^/L)	202.00 (164.00, 242.00)	201.50 (160.50, 242.00)	208.00 (177.00, 239.50)	0.135
NEUT(×10^9^/L)	3.70 (3.10, 4.80)	3.60 (3.08, 4.70)	4.00 (3.20, 5.27)	0.050
LYMPH(×10^9^/L)	1.80 (1.40, 2.20)	1.70 (1.40, 2.20)	1.90 (1.40, 2.30)	0.191
MONO(×10^9^/L)	0.40 (0.30, 0.50)	0.40 (0.30, 0.50)	0.40 (0.30, 0.50)	0.442
FBG(mmol/L)	8.12 (6.72, 10.91)	8.07 (6.72, 11.00)	8.57 (6.73, 10.30)	0.748
PBG(mmol/L)	16.31 (12.75, 20.31)	16.45 (12.63, 20.44)	15.77 (13.13, 20.19)	0.661
HbA1c(%)	8.60 (7.20, 10.30)	8.40 (7.20, 10.30)	9.08 (6.95, 10.28)	0.427
FCP(μg/L)	0.74 (0.56, 1.03)	0.76 (0.57, 1.04)	0.68 (0.47, 0.97)	0.133
FINS(μIU/ml)	9.60 (5.85, 14.10)	9.62 (6.21, 14.41)	9.10 (5.21, 13.80)	0.163
TG(mmol/L)	1.54 (1.08, 2.33)	1.54 (1.07, 2.31)	1.58 (1.12, 2.34)	0.416
TC(mmol/L)	4.73 (3.95, 5.52)	4.71 (3.95, 5.45)	4.76 (3.76, 5.95)	0.577
Cr(μmol/L)	65.90 (55.62, 81.85)	65.50 (55.05, 81.93)	66.50 (57.95, 81.28)	0.994
UA(mmol/L)	325.50 (269.00, 383.75)	324.50 (268.00, 378.50)	331.50 (272.25, 393.50)	0.655
HDL-C(mmol/L)	0.93 (0.80, 1.07)	0.92 (0.79, 1.07)	0.96 (0.86, 1.06)	0.381
LDL-C(mmol/L)	2.43 (1.86, 2.95)	2.44 (1.87, 2.94)	2.41 (1.87, 2.99)	0.778
ALT(U/L)	22.50 (15.00, 34.00)	23.00 (15.00, 34.00)	20.00 (14.25, 33.75)	0.399
AST(U/L)	23.00 (18.00, 30.00)	23.00 (19.00, 30.25)	20.00 (17.00, 30.00)	0.069
ALP(U/L)	76.00 (60.25, 93.00)	75.95 (60.00, 92.25)	78.00 (61.00, 100.75)	0.292
GGT(U/L)	28.00 (19.00, 48.00)	28.00 (19.00, 47.25)	29.00 (20.25, 48.75)	0.763
LDH(U/L)	164.50 (147.00, 188.75)	165.50 (148.00, 189.25)	159.50 (146.25, 186.00)	0.304
K(mmol/L)	3.95 (3.68, 4.21)	3.95 (3.69, 4.23)	3.95 (3.68, 4.15)	0.221
Na(mmol/L)	138.95 (136.93, 140.60)	138.95 (137.00, 140.53)	138.95 (136.90, 141.17)	0.752
Ca(mmol/L)	2.26 (2.18, 2.33)	2.26 (2.18, 2.34)	2.25 (2.18, 2.33)	0.957
Pi(mmol/L)	1.12 (1.02, 1.23)	1.11 (1.02, 1.20)	1.16 (1.03, 1.25)	0.069
Mg(mmol/L)	0.80 (0.74, 0.84)	0.80 (0.74, 0.84)	0.79 (0.75, 0.84)	0.655
AFP(ng/ml)	2.20 (1.70, 3.00)	2.20 (1.67, 3.00)	2.30 (1.72, 3.00)	0.577
CA125(U/ml)	10.35 (7.90, 13.60)	11.00 (7.75, 14.30)	11.35 (8.85, 15.00)	0.522
CA199(U/ml)	10.30 (5.40, 22.15)	10.20 (5.20, 20.47)	11.15 (6.05, 28.95)	0.088
CEA(ng/ml)	2.70 (1.90, 3.80)	2.60 (1.90, 3.80)	2.80 (2.10, 3.58)	0.572
MHR	0.41 (0.30, 0.56)	0.41 (0.30, 0.57)	0.41 (0.31, 0.51)	0.969
NHR	4.07 (3.05, 5.62)	4.00 (3.01, 5.44)	4.45 (3.17, 6.10)	0.123
PHR	213.42 (164.58, 281.84)	209.27 (162.45, 281.07)	228.48 (168.74, 283.13)	0.326
LHR	1.86 (1.36, 2.58)	1.84 (1.36, 2.58)	1.91 (1.40, 2.62)	0.466
TyG	1.89 (1.43, 2.36)	1.90 (1.43, 2.36)	1.88 (1.43, 2.35)	0.748
CHG	3.04 (2.72, 3.34)	3.06 (2.73, 3.34)	2.98 (2.65, 3.31)	0.479
WHTR	0.65(0.65, 0.66)	0.66(0.64, 0.66)	0.65(0.63, 0.65)	0.875
METS-IR	2.40(2.36, 2.62)	2.42(2.38, 2.57)	2.41(2.39, 2.41)	0.118
METS-VF	6.95(6.47, 7.12)	6.89(6.48, 7.23)	6.82(6.44, 7.13)	0.091

Data are shown as median with interquartile range (IQR) for continuous variables and number with percentage for categorical variables. Abbreviations: BMI, body mass index; SFA-L3, subcutaneous fat area of the third lumbar vertebra; VFA-L3, visceral fat area of the third lumbar vertebra; CSA-L3 cross sectional area of the third lumbar vertebra; SMI-L3, skeletal muscle index of the third lumbar vertebra; RBC, red blood cell count; HGB, hemoglobin; PLT, platelet count; NEUT, neutrophil count; LYMPH, lymphocyte count; MONO, monocyte count; FBG, fasting plasma glucose; PBG, postprandial blood glucose; HbA1c, haemoglobin A1c; FCP, fasting c peptide; FINS, fasting insulin; TG, triglycerides; TC, total cholesterol; Cr, creatinine; UA, uric acid; HDLC, high-density lipoprotein cholesterol; LDL-C, low-density lipoprotein cholesterol; ALT, alanine aminotransferase; ALP, alkaline phosphatase; AST, aspartate aminotransferase; GGT, Gamma glutamyl transferase; LDH, lactate dehydrogenase; Na, natrium; Ca, calcium; Pi, phosphorus; Mg, magnesium; AFP alpha-fetoprotein; CA125, carbohydrate antigen 125; CA199, Carbohydrate Antigen 19-9; CEA, carcinoembryonic antigen; MHR, Monocyte-to-HDL Ratio; NHR, Neutrophil-to-HDL Ratio; PHR, Platelet-to-HDL Ratio; LHR, Lymphocyte-to-HDL Ratio; TyG, Triglyceride-Glucose Index; CHG, Cholesterol-Glucose Index; WHTR, waist-to-height ratio;METS-IR, metabolic score for insulin resistance; METS-VF, metabolic score for visceral fat.

Within the training set, numerous variables differed significantly between the OP and non-OP groups (all P < 0.05), as detailed in **Table 2**. These significant variables encompassed demographics (age, sex), imaging findings (fatty liver, pancreatic steatosis, SMI-L3), routine laboratory tests (HGB, PLT, LYMPH, NEUT, RBC, UA, Ca, Pi), lipid and glucose metabolism markers (TC, TG, HDL-C, LDL-C, HbA1c, FCP, FINS, ALP, ALT), tumor markers (CA199, CEA), and derived indices (PHR, LHR, TyG, METS-VF).

**Table 2:** Results of univariate analysis.

Variables	Total (n = 682)	Non-Osteoporosis (n = 338)	Osteoporosis (n = 344)	P
Gender(%)				0.001
Male	420 (61.58)	238 (70.41)	182 (52.91)	
Female	262 (38.42)	100 (29.59)	162 (47.09)	
IPFD(%)				0.010
Yes	482 (70.67)	218 (64.50)	264 (76.74)	
No	200 (29.34)	120 (35.50)	80 (23.26)	
FLD(%)				0.033
Yes	234 (34.31)	134 (39.64)	100 (29.07)	
No	448 (65.69)	204(60.36)	244 (70.93)	
Age	60.00 (48.75, 69.00)	66.00 (43.00, 59.00)	75.00 (61.00, 74.00)	<.001
BMI(kg/m2)	24.60 (22.50, 27.33)	24.80 (22.70, 27.70)	24.39 (22.20, 27.00)	0.078
SFA-L3(cm2)	89.85 (64.90, 130.85)	89.90 (62.70, 134.30)	89.50 (66.20, 126.30)	0.967
VFA-L3(cm2)	133.69 (95.43, 180.45)	133.90 (98.10, 178.60)	132.90 (92.35, 182.65)	0.990
CSA-L3(cm2)	41.30 (35.27, 50.73)	45.80 (37.80, 55.60)	38.40 (33.25, 45.35)	<.001
SMI -L3(cm2/m2)	34.75 (30.51, 40.49)	36.63 (32.81, 42.76)	32.72 (29.39, 37.36)	<.001
RBC(×10^9^/L)	4.47 (4.07, 4.87)	4.72 (4.38, 5.10)	4.21 (3.84, 4.57)	<.001
HGB(×10^9^/L)	136.00 (122.00, 149.00)	144.00 (133.00, 155.00)	127.00 (114.50, 141.00)	<.001
PLT(×10^9^/L)	201.50 (160.50, 242.00)	211.00 (170.00, 250.00)	202.00 (164.00, 242.00)	0.124
NEUT(×10^9^/L)	3.60 (3.08, 4.70)	3.60 (3.10, 4.50)	3.95 (3.05, 4.96)	0.047
LYMPH(×10^9^/L)	1.70 (1.40, 2.20)	1.90 (1.50, 2.20)	1.70 (1.20, 2.00)	<.001
MONO(×10^9^/L)	0.40 (0.30, 0.50)	0.40 (0.30, 0.40)	0.40 (0.30, 0.50)	0.217
FBG(mmol/L)	8.07 (6.72, 11.00)	7.90 (6.69, 11.00)	8.25 (6.88, 10.97)	0.656
PBG(mmol/L)	16.45 (12.63, 20.44)	15.76 (12.74, 20.32)	17.18 (12.61, 20.48)	0.314
HbA1c(%)	8.40 (7.20, 10.30)	8.80 (7.20, 10.60)	8.30 (7.15, 10.20)	0.033
FCP(μg/L)	0.76 (0.57, 1.04)	0.75 (0.56, 0.99)	0.88 (0.57, 1.13)	<.001
FINS(μIU/ml)	9.62 (6.21, 14.41)	11.70 (6.70, 14.60)	10.90 (6.03, 13.60)	0.319
TG(mmol/L)	1.54 (1.07, 2.31)	1.74 (1.10, 2.49)	1.40 (1.02, 1.96)	0.013
TC(mmol/L)	4.71 (3.95, 5.45)	4.81 (4.13, 5.52)	4.61 (3.67, 5.43)	0.040
Cr(μmol/L)	65.50 (55.05, 81.93)	65.00 (54.70, 80.90)	66.50 (55.45, 82.85)	0.599
UA(mmol/L)	324.50 (268.00, 378.50)	338.00 (273.00, 376.00)	323.00 (266.50, 389.00)	0.004
HDL-C(mmol/L)	0.92 (0.79, 1.07)	0.91 (0.79, 1.05)	0.99 (0.79, 1.11)	0.036
LDL-C(mmol/L)	2.44 (1.87, 2.94)	2.46 (2.08, 3.01)	2.31 (1.71, 2.88)	0.016
ALT(U/L)	23.00 (15.00, 34.00)	24.00 (17.00, 37.00)	22.00 (14.00, 29.00)	0.105
AST(U/L)	23.00 (19.00, 30.25)	23.00 (19.00, 32.00)	23.00 (19.00, 28.50)	0.922
ALP(U/L)	75.95 (60.00, 92.25)	74.00 (58.00, 91.00)	87.00 (64.00, 93.50)	<.001
GGT(U/L)	28.00 (19.00, 47.25)	30.00 (21.00, 47.00)	26.00 (18.00, 49.00)	0.228
LDH(U/L)	165.50 (148.00, 189.25)	164.00 (148.00, 190.00)	167.00 (148.50, 187.50)	0.764
K(mmol/L)	3.95 (3.69, 4.23)	3.94 (3.68, 4.21)	3.96 (3.70, 4.26)	0.988
Na(mmol/L)	138.95 (137.00, 140.53)	138.80 (136.90, 140.40)	139.20 (137.10, 140.60)	0.218
Ca(mmol/L)	2.26 (2.18, 2.34)	2.27 (2.20, 2.35)	2.25 (2.17, 2.31)	0.527
Pi(mmol/L)	1.11 (1.02, 1.20)	1.13 (1.03, 1.21)	1.12 (0.98, 1.20)	0.919
Mg(mmol/L)	0.80 (0.74, 0.84)	0.80 (0.74, 0.84)	0.80 (0.75, 0.84)	0.447
AFP(ng/ml)	2.20 (1.67, 3.00)	2.20 (1.60, 3.00)	2.20 (1.70, 3.00)	0.824
CA125(U/ml)	10.00 (7.75, 13.30)	10.40 (7.80, 13.40)	9.60 (7.70, 13.30)	0.665
CA199(U/ml)	10.20 (5.20, 20.47)	10.10 (5.50, 20.70)	11.14 (5.95, 20.05)	0.427
CEA(ng/ml)	2.60 (1.90, 3.80)	2.40 (1.80, 3.90)	2.90 (2.00, 3.80)	0.003
MHR	0.41 (0.30, 0.57)	0.41 (0.31, 0.53)	0.41 (0.29, 0.60)	0.756
NHR	4.00 (3.01, 5.44)	4.06 (3.26, 5.25)	3.80 (2.83, 5.67)	0.676
PHR	209.27 (162.45, 281.07)	223.71 (175.89, 301.08)	217.50 (149.43, 258.21)	0.525
LHR	1.84 (1.36, 2.58)	2.08 (1.62, 2.74)	1.58 (1.20, 2.38)	<.001
TyG	1.90 (1.43, 2.36)	2.01 (1.45, 2.43)	1.78 (1.38, 2.26)	0.033
CHG	3.06 (2.73, 3.34)	3.08 (2.74, 3.39)	3.05 (2.72, 3.31)	0.171
WHTR	0.66(0.64, 0.66)	0.63(0.63, 0.63)	0.66(0.66, 0.69)	0.756
METS-IR	2.42(2.38, 2.47)	2.40(2.40, 2.47)	2.41(2.39, 2.43)	0.325
METS-VF	6.89(6.48, 7.23)	6.50(6.49, 6.51)	6.77(6.52, 7.11)	<.001

Data are shown as median with interquartile range (IQR) for continuous variables and number with percentage for categorical variables. Abbreviations: BMI, body mass index; SFA-L3, subcutaneous fat area of the third lumbar vertebra; VFA-L3, visceral fat area of the third lumbar vertebra; CSA-L3 cross sectional area of the third lumbar vertebra; SMI-L3, skeletal muscle index of the third lumbar vertebra; RBC, red blood cell count; HGB, hemoglobin; PLT, platelet count; NEUT, neutrophil count; LYMPH, lymphocyte count; MONO, monocyte count; FBG, fasting plasma glucose; PBG, postprandial blood glucose; HbA1c, haemoglobin A1c; FCP, fasting c peptide; FINS, fasting insulin; TG, triglycerides; TC, total cholesterol; Cr, creatinine; UA, uric acid; HDLC, high-density lipoprotein cholesterol; LDL-C, low-density lipoprotein cholesterol; ALT, alanine aminotransferase; ALP, alkaline phosphatase; AST, aspartate aminotransferase; GGT, Gamma glutamyl transferase; LDH, lactate dehydrogenase; Na, natrium; Ca, calcium; Pi, phosphorus; Mg, magnesium; AFP alpha-fetoprotein; CA125, carbohydrate antigen 125; CA199, Carbohydrate Antigen 19-9; CEA, carcinoembryonic antigen; MHR, Monocyte-to-HDL Ratio; NHR, Neutrophil-to-HDL Ratio; PHR, Platelet-to-HDL Ratio; LHR, Lymphocyte-to-HDL Ratio; TyG, Triglyceride-Glucose Index; CHG, Cholesterol-Glucose Index; WHTR, waist-to-height ratio;METS-IR, metabolic score for insulin resistance; METS-VF, metabolic score for visceral fat.

### Feature selection

From the 22 variables found to be significant in univariate analysis (**Table 2**), further feature selection was performed using LASSO regression and the Boruta algorithm. LASSO regression, with an optimal lambda (λ) of 0.034, identified 8 key features, while the Boruta algorithm identified 9 key features. The intersection of these two methods yielded seven robust core predictors: Age, HGB, NEUT, UA, LHR, SMI-L3, and METS-VF (**Figure 1**). All selected predictors showed low multicollinearity, with Pearson correlation coefficients r < 0.80 and Variance Inflation Factor (VIF) values below 10.

### Model development and evaluation

We evaluated the performance of seven ML algorithms: XGBoost, LR, LightGBM, GBDT, MLP, GNB, and SVM. The model performance metrics are summarized in **Table 3**.

**Table 3:** The prediction performance of each model in different data sets, including AUC, AUPRC, accuracy, sensitivity, specificity, precision, and F1 score.

Model	AUC(95%CI)	AUPRC(95%CI)	Accuracy	Sensitivity	Specificity	Precision	F1 Score
Training set							
XGBoost	0.877 (0.830-0.923)	0.865 (0.825–0.904)	0.817	0.854	0.780	0.795	0.823
Logistic	0.862 (0.811-0.912)	0.852 (0.825–0.879)	0.784	0.910	0.659	0.727	0.808
LightGBM	0.839 (0.788-0.891)	0.775 (0.735–0.814)	0.789	0.896	0.682	0.736	0.808
GBDT	0.834 (0.783-0.886)	0.777 (0.713–0.841)	0.791	0.873	0.710	0.750	0.806
GNB	0.849 (0.797-0.902)	0.833 (0.794–0.872)	0.791	0.877	0.706	0.747	0.807
MLP	0.755 (0.692-0.819)	0.728 (0.529–0.926)	0.723	0.783	0.664	0.72	0.742
SVM	0.855 (0.805-0.906)	0.844 (0.786–0.903)	0.791	0.797	0.785	0.793	0.793
Test set							
XGBoost	0.839 (0.772-0.885)	0.785 (0.72–0.849)	0.788	0.884	0.748	0.775	0.811
Logistic	0.829 (0.772-0.885)	0.813 (0.788–0.838)	0.754	0.858	0.650	0.710	0.776
LightGBM	0.799 (0.740-0.857)	0.735 (0.625–0.844)	0.779	0.896	0.604	0.727	0.802
GBDT	0.793 (0.735-0.851)	0.724 (0.602–0.845)	0.779	0.882	0.678	0.732	0.801
GNB	0.818 (0.759-0.876)	0.787 (0.751–0.823)	0.756	0.849	0.664	0.718	0.777
MLP	0.764 (0.700-0.828)	0.752 (0.702–0.802)	0.701	0.745	0.654	0.685	0.709
SVM	0.841 (0.789-0.894)	0.825 (0.718–0.932)	0.779	0.783	0.776	0.775	0.679
External validation set							
XGBoost	0.911 (0.879-0.943)	0.900 (0.895–0.906)	0.856	0.848	0.842	0.841	0.780
Logistic	0.867 (0.826-0.908)	0.850 (0.843–0.858)	0.832	0.809	0.808	0.833	0.640
LightGBM	0.788 (0.743-0.833)	0.710 (0.705–0.715)	0.833	0.719	0.747	0.840	0.574
GBDT	0.793 (0.748-0.838)	0.720 (0.713–0.727)	0.834	0.752	0.767	0.829	0.585
GNB	0.843 (0.798-0.888)	0.824 (0.817–0.831)	0.804	0.785	0.783	0.806	0.588
MLP	0.813 (0.765-0.861)	0.706 (0.700–0.804)	0.752	0.761	0.755	0.765	0.512
SVM	0.828 (0.783-0.874)	0.804 (0.777–0.831)	0.807	0.732	0.745	0.802	0.538

In the training set, most models achieved AUC values exceeding 0.83. The XGBoost model yielded the highest AUC (0.877) and outperformed the other models in accuracy (0.817), precision (0.795), and F1 score (0.823), while maintaining good specificity (0.780) and sensitivity (0.854). The SVM model exhibited balanced performance, with an AUC of 0.855, sensitivity of 0.797, accuracy of 0.791, precision of 0.793, and F1 score of 0.793. Although the LR model achieved the highest sensitivity (0.910) and the second-highest AUC (0.862), its accuracy (0.784), specificity (0.659), and precision (0.727) were relatively low.

In the test set, the XGBoost model maintained stable performance, with an AUC of 0.839, accuracy of 0.788, sensitivity of 0.884, and precision of 0.755, outperforming the other models. Although SVM achieved a slightly higher AUC (0.841), its sensitivity (0.797) and F1 score (0.679) were low. The LightGBM model exhibited the highest sensitivity (0.865) but the lowest specificity (0.604).

**Figure 3:**
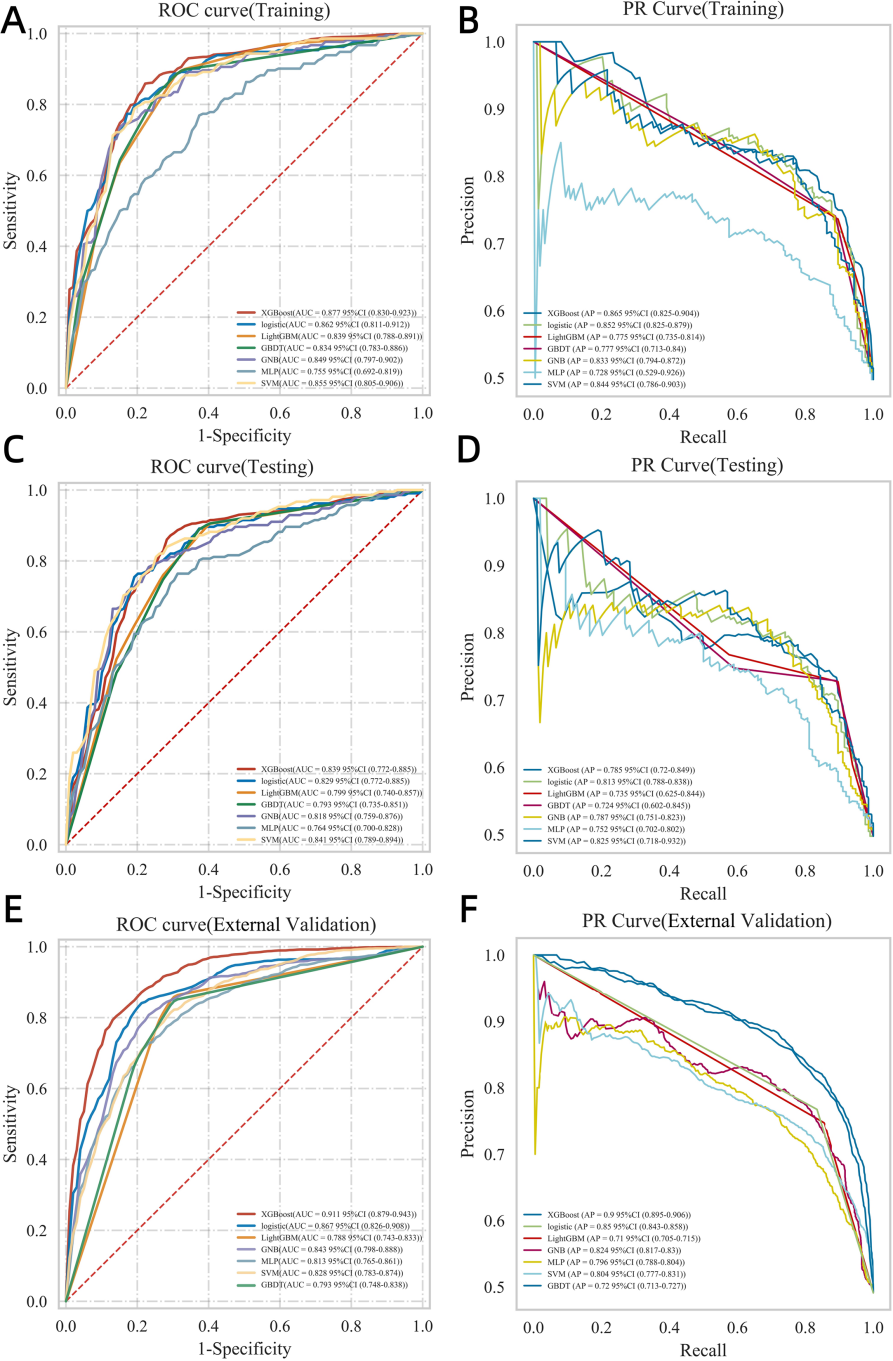
Model performance evaluation: **(A, B)**. Training Set: ROC Curves (Left) and Precision–Recall Curves (Right). **(C, D)**. Internal Testing Set: ROC Curves (Left) and Precision–Recall Curves (Right). **(E, F)** External Validation Set: ROC Curves (Left) and Precision–Recall Curves (Right).

In the external validation set, the XGBoost model outperformed the others, with an AUC of 0.911, accuracy of 0.856, sensitivity of 0.847, specificity of 0.842, precision of 0.841, and F1 score of 0.780. The ROC curves and precision-recall curves for each dataset are shown in **Figure 3**. Following a comprehensive evaluation across all datasets, the XGBoost model was ultimately selected as the optimal classification model. The XGBoost model maintained AUC values above 0.83 across the training, test, and external validation sets, with F1 scores consistently ranging from 0.780 to 0.823, and exhibited no signs of overfitting.

Additionally, the AUPRC values further supported the XGBoost model’s superior performance: 0.865 (95% CI: 0.825–0.904) in the training set, 0.785 (95% CI: 0.72–0.849) in the test set, and 0.9 (95% CI: 0.895–0.906) in the external validation set, as illustrated in the precision-recall curves (**Figures 3B, D, F**).

**Figure 4:**
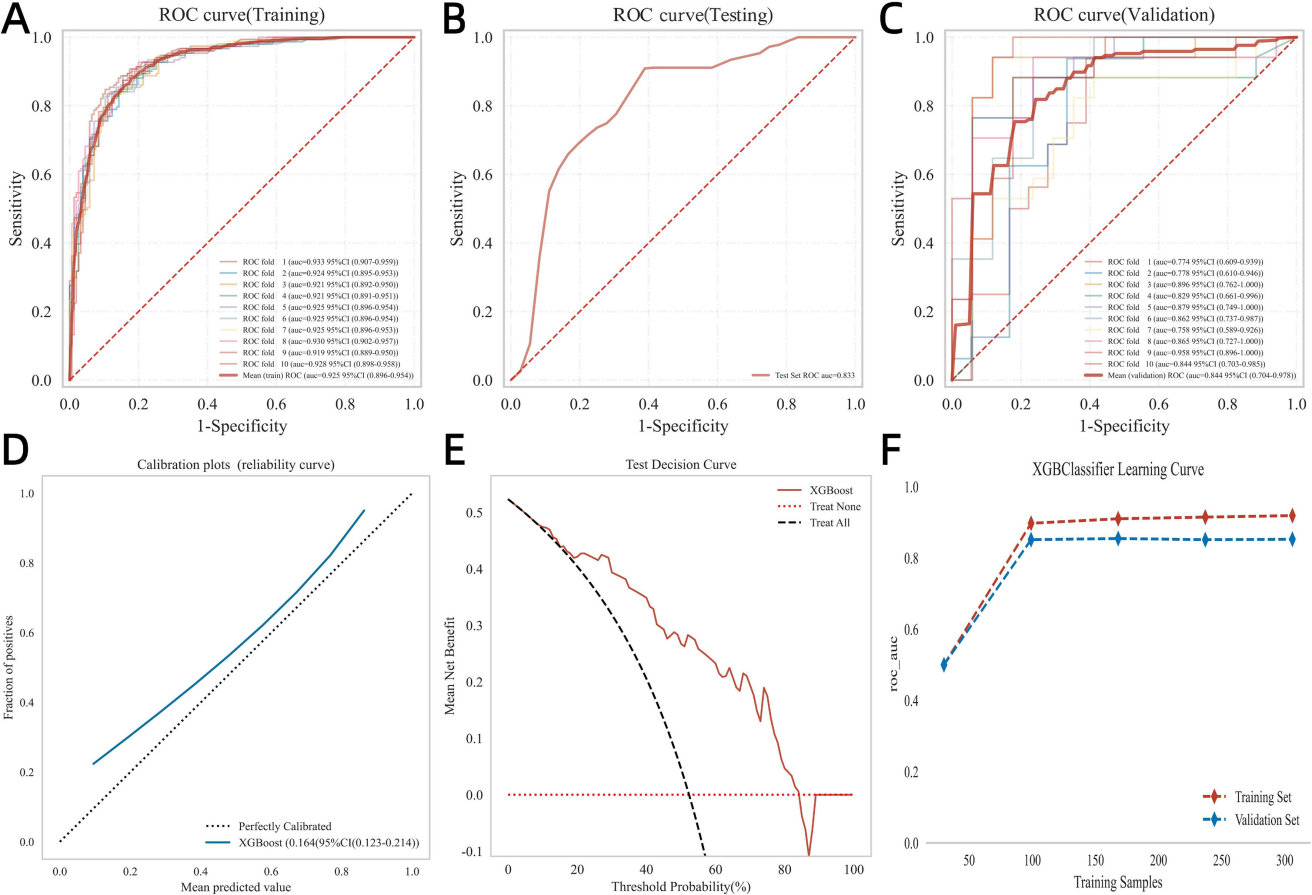
Construction and assessment of extreme gradient boosting (XGBoost) classification prediction model. **(A-C)** The ROC curves of XGBoost using the 10-fold nested cross-validation on the training set **(A)**, test set **(B)**, and validation set **(C, D)** Calibration plots (reliability curves) for the XGBoost model, with the dashed diagonal line representing perfect calibration. **(E)** Decision curve analysis graph showing the net benefit against threshold probabilities based on decisions from model outputs. **(F)** Machine learning curve.

The results of the 10-fold nested cross-validation further confirmed the excellent predictive performance and superior generalization ability of the XGBoost model: the mean AUC was 0.925 for the training set, 0.833 for the test set, and 0.844 for the validation set (**Figures 4A–C**). The calibration curves (**Figure 4D**) demonstrated good agreement between the predicted probabilities from the XGBoost model and the actual probability of osteoporosis, with the calibration curve closely approximating the 45-degree diagonal reference line across all datasets. The decision curves (**Figure 4E**) confirmed the clinical utility of the model across a range of threshold probabilities.

### Model interpretation and web application

**Figure 5:**
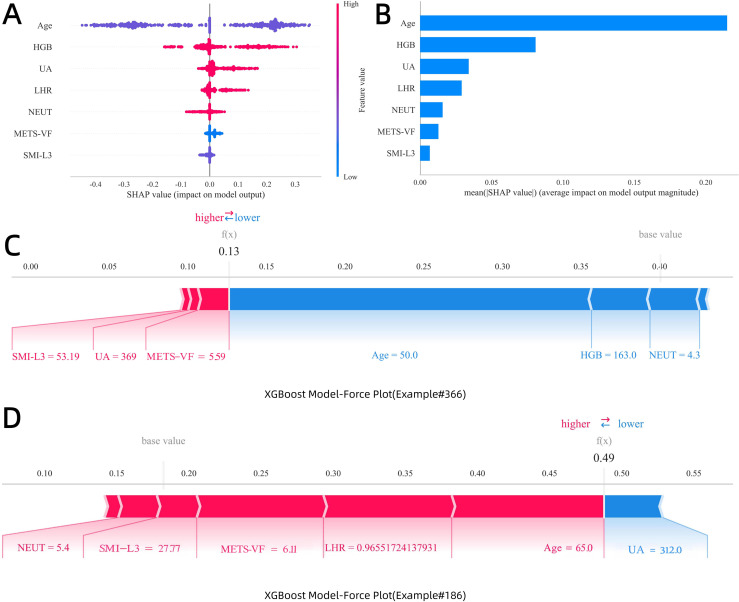
SHAP interpretation of the XGboost model: **(a)** SHAP summary plot, Positive SHAP values (right) boost the predicted probability of osteoporosis, while negative ones (left) lower it. Features are colored from low (blue) to high (red), illustrating how different value ranges affect predictions. **(b)** Feature importance ranking, reflecting their average influence on predicting osteoporosis. Higher values indicate greater feature importance. **(c, d)** SHAP force plots for individual predictions: individual contributions from T2DM patients with osteoporosis **(c, d)** without osteoporosis. The baseline (model’s average output) serves as a reference. Positive contributions (red) raise the predicted osteoporosis probability, while negative ones (blue) reduce it.

To elucidate the decision-making process of the XGBoost model, we applied the SHAP framework. The bar plot of mean absolute SHAP values (**Figure 5A**) and the SHAP summary plot (**Figure 5B**) ranked feature importance, identifying age, HGB, UA, LHR, NEUT, METS-VF, and SMI-L3 as the most influential predictors (in descending order). The force plots (**Figures 5C, D**) illustrate the contribution of individual features to the prediction for representative patients with and without osteoporosis. These findings highlight the critical roles of age-related bone loss, potential chronic hypoxia indicated by anemia, and the oxidative stress and inflammation associated with altered uric acid levels in the pathogenesis of osteoporosis in T2DM.

To facilitate clinical application, we developed a web-based prediction tool based on the XGBoost model, accessible at http://www.xsmartanalysis.com/model/list/predict/model/html?mid=31500&symbol=6Qexq1770695388TpNF6/(interface shown in **Supplementary Figure 3**). This tool enables real-time, individualized osteoporosis risk assessment for T2DM patients by inputting the seven key predictors. However, this web application should be regarded as a proof-of-concept tool that still requires prospective clinical validation and usability testing before its clinical utility can be established.

## Discussion

This study distinguishes itself from prior work by developing a multimodal diagnostic classification model for osteoporosis risk in patients with T2DM that uniquely integrates CT-derived body composition parameters (SMI-L3 and METS-VF) with routine clinical and laboratory data, and systematically evaluates seven machine learning algorithms to identify the optimal predictive framework. The model incorporates demographic characteristics, cross-sectional imaging information at the level of the third lumbar vertebra, laboratory test indicators, and multiple derived composite indices. It should be noted that the term “multimodal” in this context refers to the integration of data from distinct information types—demographics, laboratory tests, CT-derived body composition parameters, and composite metabolic indices—rather than the combination of multiple imaging modalities (e.g., DXA, CT, and MRI). While this usage aligns with the broader definition of multimodality increasingly adopted in clinical prediction modeling, it should be distinguished from multimodal imaging approaches. Following a comprehensive evaluation of model performance metrics, the XGBoost model demonstrated superior performance. We optimized this model through automatic hyperparameter tuning and further assessed its accuracy and robustness using cross-validation with nested loops, complemented by calibration curves and decision curve analysis (DCA). Furthermore, an exploratory analysis using SHAP values was conducted, which intuitively revealed, through quantitative means, the contribution and importance of seven core predictors in forecasting the occurrence of osteoporosis.

The adverse effects of T2DM on bone physiology are likely multifactorial, involving mechanisms such as aging, inadequate glycemic control, abnormal metabolic states, electrolyte disturbances, and the body’s inflammatory response status, all of which may contribute to osteoporosis. Therefore, building upon previous research, we incorporated as many clinically accessible risk factors for osteoporosis in T2DM patients as possible. While our model accurately identifies osteoporosis defined by DXA T-score, the well-documented limitation of DXA in T2DM—namely its underestimation of fracture risk due to impaired bone quality and AGE accumulation—must be acknowledged. However, the model’s strength lies precisely in its inclusion of features beyond BMD that contribute to diabetic bone fragility. METS-VF reflects visceral adiposity and inflammatory adipokine dysregulation; SMI-L3 captures sarcopenia-related myokine imbalance; and LHR indicates systemic inflammation. These factors are independent contributors to fracture susceptibility and are not captured by DXA. Thus, while the model currently classifies DXA-defined osteoporosis, its multimodal composition may implicitly capture residual fracture risk, offering potential clinical value beyond T-score replication. Prospective validation against incident fracture endpoints is nevertheless required. We also included multiple derived composite variables, calculated based on demographic features and laboratory indicators, in the initial variable pool for core predictor selection. A multi-step feature selection process was employed to address multicollinearity among the numerous variables. Ultimately, seven core predictors were identified: Age, HGB, UA, LHR, NEUT, METS-VF, and SMI-L3. These were used for the final machine learning modeling.

Age has consistently been recognized as a significant influencing factor for osteoporosis in T2DM patients. Aging disrupts bone homeostasis by impairing osteoblast function and accelerating osteoclast activation (36). A study by Wu et al. focusing on a Chinese cohort with T2DM also demonstrated that the prevalence of osteoporosis escalates markedly with advancing age, reporting a rate of 28.6% in patients aged 50–59 years, which increased to 44.1% in those aged 60–69 years, and further to 57.1% in patients over 70 years. This finding aligns with our results and corroborates the widely accepted view in the field that bone mass declines with age after reaching its peak (2). Reduced HGB levels, indicative of anemia, imply a state of hypoxia in the body. Chronic hypoxia resulting from anemia can activate hypoxia-inducible factors (e.g., HIF-1α, HIF-2α), which in turn stimulate osteoclast activity, thereby impairing bone repair capacity (37–39). Uric acid plays a complex role in bone metabolism, particularly in patients with T2DM. While physiological levels of UA may offer antioxidant benefits, elevated UA levels in T2DM patients are often associated with adverse metabolic states that can exacerbate bone loss and increase fracture risk (40). From a metabolic perspective, although UA possesses antioxidant properties, its pro-inflammatory effects may counteract its protective role (40). LHR is primarily considered a comprehensive index reflecting immune status and metabolic function. A study based on NHANES data from 2011–2018 indicated that LHR is significantly negatively correlated with bone mineral density (BMD) in the lumbar spine, trunk, and total body. Lymphocytes can directly promote osteoclast differentiation by secreting factors like RANKL, leading to increased bone resorption. Conversely, HDL-C not only has anti-atherosclerotic effects but also promotes bone formation and inhibits bone resorption. Therefore, when HDL-C decreases, leading to an increased LHR, the protective effect on BMD is weakened (41). An elevated LHR also signifies an activated immune system, often accompanied by increased levels of inflammatory cytokines such as IL-6 and TNF-α, which promote osteoclast differentiation, accelerate BMD loss, and increase fracture risk – a finding consistent with previous research (42). Regarding the selection of NEUT over NHR, this was directly determined by the multi-step feature selection procedure. In the univariate analysis (**Table 2**), NEUT showed a statistically significant association with osteoporosis (P = 0.047), whereas NHR did not reach significance (P = 0.676), and thus NHR was excluded prior to the LASSO/Boruta step. Consequently, only NEUT entered the LASSO and Boruta feature selection pipeline, where it was consistently retained as a robust core predictor (**Figure 1**). This data-driven selection is biologically plausible, as neutrophils play a direct role in bone metabolism—capable of both promoting bone resorption and exerting protective effects—whereas NHR reflects systemic inflammation and nutritional status more indirectly. It is noteworthy that although our model shares several core variables (Age, HGB, UA, LHR, NEUT) with the study by Wei et al., the inclusion of METS-VF and SMI-L3 – two imaging biomarkers comprehensively reflecting metabolism and body composition – may have contributed to the model’s enhanced discriminative performance. Compared with the model by Wei et al., which used only laboratory and demographic variables and achieved a training AUC of 0.835, our model incorporating these additional imaging biomarkers achieved a training AUC of 0.877 and an external validation AUC of 0.911. However, without a direct ablation comparison within the same modeling framework, this attribution remains speculative, and future studies should systematically evaluate the incremental value of these variables through dedicated ablation analyses (40, 41, 43, 44).

Studies by Zhang B et al. have confirmed that a reduction in the skeletal muscle index (SMI) is a significant risk factor for the development of osteoporosis in patients with diabetes mellitus (45). This finding aligns closely with the “sarcopenia-insulin resistance vicious cycle” theory proposed by Cleasby et al., which posits that decreased muscle mass exacerbates abnormalities in glucose metabolism (46). However, the crosstalk between muscle and bone extends far beyond glucose metabolism. From a molecular perspective, skeletal muscle serves not only as the primary target for insulin-mediated glucose uptake but also as an active endocrine organ. When muscle mass declines, the following pathological changes occur: 1) Reduced expression of GLUT4 transporters, leading to a 30%-40% decrease in glucose uptake capacity by skeletal muscle; 2) Impaired mitochondrial function in myocytes, with reduced β-oxidation capacity contributing to lipotoxicity; 3) Most critically, a disruption in the secretion profile of myokines, which constitutes a core component of the muscle-bone endocrine axis. Specifically, levels of protective myokines that promote osteoblast differentiation and inhibit osteoclast activity, such as irisin, are significantly reduced, while the secretion of pro-inflammatory myokines like IL-6 is increased (47, 48). Inflammatory factors such as IL-6 can directly promote osteoclast differentiation and bone resorption by activating the receptor activator of nuclear factor-κB ligand (RANKL) pathway, thereby accelerating bone loss. This imbalance in the myokine regulatory network directly disrupts the homeostasis of the “muscle-bone” unit, further exacerbating degenerative changes. Clinically, muscle loss and osteoporosis frequently co-occur, forming a condition known as “osteosarcopenia, “ suggesting they share common pathophysiological foundations, including aging, hormonal imbalance, chronic inflammation, and nutritional metabolic disorders.

These mechanisms, driven by insulin resistance, oxidative stress, and the accumulation of advanced glycation end products (AGEs), interact to form a complex “metabolic-inflammatory-bone loss” network, ultimately leading to decreased bone mineral density and increased fracture risk. Our study is the first to incorporate METS-VF as a featured variable in a predictive model for osteoporosis in T2DM patients. METS-VF, a novel metabolic indicator reflecting an individual’s risk of insulin resistance, visceral fat accumulation, and overall metabolic dysregulation, offers superior capability in capturing metabolically active visceral fat compared to traditional measurement methods. METS-VF assesses osteoporosis risk more sensitively and clinically relevantly than conventional obesity indicators (49, 50). Researchers including Sharma, P., et al., utilizing restricted cubic splines (RCS) and threshold effect analysis, discovered a significant non-linear association between METS-VF and lumbar spine bone mineral density (LS BMD), identifying a notable inflection point at METS-VF = 5.47. This suggests that the metabolic burden of visceral fat exerts a negative impact on BMD once it surpasses a specific threshold. This phenomenon may be related to the protective effects of mechanical load or adipose-derived estrogen on bone tissue at lower fat levels (51). However, once visceral fat accumulation exceeds the physiological regulatory range, it may enhance bone resorption and inhibit bone formation by inducing chronic low-grade inflammation, insulin resistance, and endocrine disorders, thereby accelerating bone loss (52, 53). Notably, through multi-step feature selection, our study retained both SMI-L3 and METS-VF in the final model, rather than choosing one over the other. Specifically, visceral adipose tissue directly and indirectly regulates bone metabolism by secreting various bioactive substances, such as adiponectin, leptin, and pro-inflammatory cytokines (e.g., TNF-α, IL-6). Adiponectin typically promotes bone formation, but its levels are downregulated in obesity, diminishing its skeletal protective effect; leptin can bidirectionally regulate bone formation and resorption via the central nervous system or through direct action on bone cells; while inflammatory cytokines like TNF-α and IL-6 can activate osteoclasts, promoting bone resorption and inhibiting bone formation, thus accelerating bone loss (54, 55). This dysregulation of adipokines constitutes a central mechanism by which visceral fat accumulation contributes to osteoporosis. This suggests that in T2DM patients, “sarcopenia” and “visceral fat dysfunction” may jointly promote the development of osteoporosis through distinct yet interconnected pathways. SMI-L3 represents the “quantity” and “quality” of skeletal muscle, serving as a “reservoir” for the body’s protein reserves and insulin sensitivity; METS-VF quantifies the “metabolic burden” of visceral fat, acting as an “amplifier” of chronic inflammation and oxidative stress. Integrating both into a single model allows for a more refined characterization of the “muscle-fat imbalance” metabolic phenotype in T2DM patients, which may have contributed to the model’s strong performance (AUC 0.911) in multi-center external validation.

Although previous studies have confirmed the important role of 25-hydroxyvitamin D [25(OH)D] in bone metabolism, it was not incorporated into our model construction (56, 57) for the following reasons: First, 25(OH)D levels are highly influenced by external factors such as season, sunlight exposure, and supplement use, exhibiting significant variability and potentially introducing confounding bias in multivariate models. Second, the variables included in this study—Age, UA, HGB, LHR, SMI-L3, and METS-VF—comprehensively reflect chronic inflammation, metabolic disorders, muscle mass, and endocrine status, factors known to biologically interact with vitamin D pathways. Notably, a bidirectional regulatory relationship exists between SMI-L3 and 25(OH)D. On one hand, the vitamin D receptor (VDR) is widely expressed in skeletal muscle cells; 1, 25-(OH)_2_D_3_ can promote myocyte differentiation and myofibril synthesis via genomic pathways, maintaining muscle strength and mass. Low 25(OH)D levels have been confirmed to be closely associated with sarcopenia, gait instability, and increased fall risk. On the other hand, muscle tissue is not only a target for vitamin D action but also part of its metabolic microenvironment—decreased muscle mass may further impair the peripheral activation and utilization efficiency of vitamin D, creating a “sarcopenia-vitamin D deficiency-bone loss” vicious cycle (58, 59). SMI-L3, as an imaging biomarker for quantitatively assessing skeletal muscle content, can objectively reflect the functional status of this interactive axis. Therefore, SMI-L3 may not only represent muscle reserve but also serve as a “functional integrative indicator” of vitamin D’s biological effects, capturing its cumulative impact on the musculoskeletal system without directly measuring 25(OH)D. Furthermore, METS-VF reflects visceral fat accumulation, which is linked to the “sequestration effect” of vitamin D in adipose tissue; hemoglobin and uric acid act as proxy indicators for chronic inflammation and oxidative stress, indirectly reflecting the systemic burden on the bone microenvironment (60, 61). In summary, although 25(OH)D was not directly included, our model, by integrating SMI-L3 and METS-VF, may partially substitute for the predictive information of vitamin D from the perspective of muscle-fat metabolism, constituting a potential advantage of this study in multimodal data integration.

This paper also employed the SHAP method to assess feature importance, which not only quantifies the contribution value of each feature but also provides a reference for clinical decision-making, thereby evaluating the independent value of variables and promoting the development of personalized medicine concepts. According to the feature importance analysis of the algorithmic model, the top three ranked features were Age, HGB, and UA, with interdependencies observed among these three variables. Lower HGB combined with higher age enhanced the model’s interpretability, consistent with current understanding of osteoporosis pathogenesis in diabetes (62).

To further contextualize the performance and contribution of our model, we compared it with several recently published diagnostic models for osteoporosis in patients with T2DM. Wei et al. developed an explainable machine learning model based on 18 clinical and laboratory variables, achieving an internal validation AUC of 0.835; however, their study lacked external validation, which may limit the assessment of its generalizability (44). Similarly, Tan et al. (63) established a multicenter risk prediction model using eight demographic and laboratory factors, reporting an external validation AUC of 0.816. Wu et al. evaluated multiple machine learning algorithms for osteoporosis prediction in a single-center T2DM cohort, with the best model achieving an AUC of 0.86 in internal testing (2). In contrast, our model exhibits several distinct advancements. First, the integration of multimodal data from distinct information types—specifically the CT-derived body composition parameters SMI-L3 and METS-VF—enables a more comprehensive capture of the pathophysiological interplay between sarcopenia, visceral adiposity, and bone metabolism, a dimension not addressed by models relying solely on demographics and laboratory tests. Second, our study design includes a rigorous, independent external validation cohort, demonstrating superior and more generalizable performance (AUC 0.911) compared to prior studies. Third, the implementation of the SHAP framework provides a transparent, quantitative explanation of individual predictions, which is critical for fostering clinical trust and facilitating personalized decision-making beyond what is offered by conventional models. These comparisons underscore that our model represents a significant step toward a more precise and clinically actionable tool for osteoporosis risk stratification in T2DM patients.

Our study has certain limitations: 1. Our study only included data from two tertiary hospitals in Zhejiang Province. Future research should aim to incorporate data from healthcare systems in other regions to conduct prospective multi-center validation, which is crucial for assessing the robustness and applicability of this model in broader clinical settings. 2. This retrospective study was inherently limited by the scope of data available in the electronic medical record system, thereby restricting our ability to obtain information on potential confounders that significantly impact bone metabolism, such as patients’ physical activity levels, daily sunlight exposure duration, calcium or vitamin D supplementation, and detailed history of glucose-lowering medication use (e.g., metformin, sulfonylureas, thiazolidinediones). Furthermore, the inclusion of CT-derived parameters (SMI-L3 and METS-VF) enhances predictive accuracy but restricts the model’s immediate applicability to the subset of patients for whom an abdominal CT scan is clinically indicated and available. While these opportunistic images provide valuable metabolic and body composition insights, they are not routinely acquired for all patients with T2DM in primary care. Future prospective studies could explore whether targeted, low-dose CT protocols might be cost-effective for combined osteoporosis and body composition screening in high-risk T2DM populations. 3. This study evaluated only seven machine learning algorithms, which may limit the scope of model performance comparison. Future studies should not be confined to generic black-box models but should focus on developing ensemble learning methods to further enhance predictive accuracy by integrating the strengths of multiple base learners. 4.The exclusion of patients currently using glucocorticoids, bisphosphonates, hormone replacement therapy, or proton pump inhibitors limits the generalizability of our model to the broader T2DM population in clinical practice, as these medications are commonly prescribed in this patient group and may significantly influence bone metabolism and osteoporosis risk. Future studies should evaluate the model’s performance in patients receiving these medications to determine its applicability in more representative clinical populations. 5. Although the absence of 25-hydroxyvitamin D [25(OH)D] from the model was justified by its high variability due to seasonal, geographic, and supplementation-related factors, as well as the potential indirect representation of vitamin D pathways through SMI-L3 and METS-VF (as discussed above), the exclusion of this key bone metabolism biomarker may limit the model’s capacity to fully capture the vitamin D–bone axis and could affect model integrity in populations with prevalent vitamin D deficiency. Future studies incorporating standardized 25(OH)D measurements are warranted to evaluate its incremental predictive value. Furthermore, introducing automated machine learning techniques represents an important exploration direction, enabling systematic searching for optimal models and hyperparameter combinations, thereby reducing manual selection bias and enhancing the model’s credibility and generalization ability in practical application scenarios.

## Conclusion

In conclusion, this study developed and validated a multimodal interpretable machine learning model that innovatively integrated the skeletal muscle index (SMI-L3) and the metabolic score for visceral fat (METS-VF) using routine clinical data. This model not only demonstrated excellent predictive performance and generalization ability in multi-center data but also, through SHAP analysis, revealed the pivotal role of muscle-fat metabolic imbalance in T2DM-related osteoporosis. This XGBoost-based model provides a proof-of-concept screening tool for osteoporosis risk stratification in T2DM patients, with the potential to support targeted DXA referral and clinical decision-making pending prospective validation, particularly in primary care settings.

## Data Availability

The raw data supporting the conclusions of this article will be made available by the authors, without undue reservation.
