# Supplementary material for: Development and validation of a multimodal interpretable machine learning model for the identification of osteoporosis in patients with type 2 diabetes mellitus: a multicenter retrospective study
Source: Front Endocrinol (Lausanne). 2026 Jul 1;17:1871923. doi: 10.3389/fendo.2026.1871923 (PMC13368566; doi:10.3389/fendo.2026.1871923)
Supplement: Supplementary file 1 [file DataSheet1.docx]

**Supplementary Materials**

**Table S1 1**

**Table S2 2**

**Table S3 3**

**Figure S1 4**

**Figure S2 5**

**Figure S3 6**

**Table S1 Baseline characteristics of patients in the external validation set**

| Variables | Total (n = 126) | Non-Osteoporosis  (n = 64) | Osteoporosis  (n = 62) | *P* |
| --- | --- | --- | --- | --- |
|  |  |  |  |  |
| Gender, n(%) |  |  |  | 0.513 |
| Male | 72 (57.14) | 34 (53.12) | 38 (61.29) |  |
| Female | 54 (42.86) | 30 (46.88) | 24 (38.71) |  |
| IPFD, n(%) |  |  |  | 0.045 |
| Yes | 48(38.10) | 30 (46.88) | 18 (29.03) |  |
| No | 78 (61.90) | 34 (53.12) | 44(70.97) |  |
| FLD, n(%) |  |  |  | 0.524 |
| Yes | 90 (71.43) | 48 (75.00) | 42 (67.74) |  |
| No | 36 (28.57) | 16 (25.00) | 20 (32.26) |  |
| Age, years | 65.00 (54.00, 72.00) | 57.00 (46.00, 65.00) | 70.00 (65.00, 75.00) | <.001 |
| BMI(kg/m^2^) | 25.60 (22.90, 28.45) | 23.95 (22.40, 27.07) | 25.70 (23.50, 29.85) | 0.091 |
| SFA-L3(cm^2^ ) | 105.90 (80.70, 154.40) | 104.80 (76.75, 143.48) | 125.90 (85.90, 154.75) | 0.032 |
| VFA-L3(cm^2^ ) | 130.30 (93.15, 179.15) | 122.80 (90.85, 172.00) | 145.25 (98.58, 189.88) | <.001 |
| CSA-L3(cm^2^ ) | 59.20 (48.20, 71.45) | 56.20 (48.20, 65.15) | 63.85 (49.23, 74.75) | 0.046 |
| SMI -L3(cm^2^/m^2^) | 35.76 (28.52, 40.94) | 37.46 (28.55, 42.14) | 33.12 (28.58, 39.05) |  |
| RBC(× 10^9^ /L) | 4.37 (4.06, 4.61) | 4.45 (4.06, 5.08) | 4.11 (4.06, 4.49) | <.001 |
| HGB(× 10^9^ /L) | 129.00 (118.25, 146.00) | 132.50 (119.75, 149.50) | 120.50 (114.00, 144.00) | <.001 |
| PLT(× 10^9^ /L) | 209.00 (149.50, 273.00) | 202.50 (141.75, 236.50) | 212.00 (156.00, 292.00) | 0.208 |
| NEUT(× 10^9^ /L) | 4.18 (3.16, 6.26) | 3.78 (3.16, 5.04) | 5.48 (3.16, 8.66) | <.001 |
| LPMPH(× 10^9^ /L) | 1.28 (0.81, 1.78) | 1.42 (0.92, 1.86) | 1.05 (0.77, 1.68) | 0.003 |
| MONO(× 10^9^ /L) | 0.40 (0.29, 0.53) | 0.39 (0.30, 0.51) | 0.41 (0.28, 0.54) | 0.945 |
| FBG(mmol/L) | 8.60 (6.83, 10.96) | 8.38 (7.35, 14.38) | 8.71 (6.71, 10.54) | 0.634 |
| PBG(mmol/L) | 17.33 (12.65, 19.07) | 14.49 (11.61, 18.20) | 18.16 (15.15, 20.10) | 0.049 |
| HbA1c(%) | 9.00 (7.30, 12.10) | 8.60 (7.38, 11.97) | 9.65 (7.20, 12.35) | 0.612 |
| FCP(μg/L) | 1.94 (1.38, 2.83) | 1.78 (1.08, 2.68) | 2.14 (1.38, 3.38) | <.001 |
| FINS(μIU/ml) | 13.00 (6.62, 30.30) | 20.51 (6.21, 38.17) | 11.79 (6.88, 21.24) | <.001 |
| TG(mmol/L) | 1.64 (1.04, 2.45) | 1.77 (1.16, 2.44 | 1.30 (1.04, 2.73) | 0.390 |
| TC(mmol/L) | 4.50 (3.35, 5.29) | 4.39 (3.40, 5.31) | 4.54 (3.12, 5.23) | 0.695 |
| Cr(μmol/L) | 80.15 (73.40, 100.18) | 81.65 (73.18, 98.05) | 79.60 (73.98, 111.83) | 0.642 |
| UA(mmol/L) | 330.90 (266.70, 412.40) | 351.20 (283.88, 397.23) | 314.20 (245.35, 453.00) | <.001 |
| HDL-C(mmol/L) | 1.01 (0.81, 1.15) | 1.04 (0.86, 1.18) | 0.98 (0.78, 1.08) | 0.189 |
| LDL-C(mmol/L) | 2.54 (1.85, 3.11) | 2.50 (1.86, 3.06) | 2.54 (1.69, 3.14) | 0.794 |
| ALT(U/L) | 23.05 (13.65, 34.97) | 22.70 (12.47, 31.98) | 25.00 (16.38, 36.73) | 0.526 |
| AST(U/L) | 22.60 (17.60, 35.00) | 21.70 (19.35, 31.38) | 22.90 (14.95, 35.00) | 0.597 |
| ALP(U/L) | 85.30 (61.50, 105.90) | 81.05 (62.08, 108.53) | 92.30 (63.35, 101.60) | <.001 |
| GGT(U/L) | 28.00 (20.60, 44.00) | 30.50 (20.50, 51.55) | 27.70 (20.60, 39.85) | 0.668 |
| LDH(U/L) | 196.30 (180.40, 236.40) | 200.60 (176.83, 243.70) | 193.90 (182.15, 231.25) | 0.522 |
| K(mmol/L) | 4.04 (3.81, 4.31) | 4.11 (3.93, 4.39) | 4.01 (3.78, 4.22) | 0.332 |
| Na(mmol/L) | 138.30 (135.50, 139.95) | 138.30 (137.00, 139.53) | 138.40 (134.15, 140.30) | 0.951 |
| Ca(mmol/L) | 2.31 (2.21, 2.39) | 2.31 (2.25, 2.39) | 2.11 (2.17, 2.41) | 0.027 |
| Pi(mmol/L) | 1.08 (0.96, 1.27) | 1.07 (0.90, 1.28) | 1.08 (0.97, 1.25) | 0.514 |
| Mg(mmol/L) | 0.83 (0.77, 0.89) | 0.84 (0.79, 0.89) | 0.83 (0.76, 0.89) | 0.695 |
| AFP(ng/ml) | 2.50 (1.74, 3.49) | 3.16 (2.09, 3.88) | 2.22 (1.44, 2.59) | 0.003 |
| CA125(U/ml) | 11.90 (8.10, 18.70) | 10.30 (7.97, 17.18) | 13.90 (10.10, 19.20) | 0.275 |
| CA199(U/ml) | 12.80 (5.90, 28.70) | 11.50 (5.65, 20.00) | 19.70 (7.18, 34.90) | <.001 |
| CEA(ng/ml) | 2.74 (1.88, 4.47) | 2.17 (1.71, 3.65) | 3.17 (2.35, 5.18) | <.001 |
| MHR | 0.40 (0.30, 0.54) | 0.40 (0.33, 0.54) | 0.39 (0.29, 0.50) | 0.398 |
| NHR | 3.75 (3.10, 4.90) | 3.70 (3.10, 4.85) | 3.81 (3.06, 4.90) | 0.897 |
| PHR | 207.34 (153.19, 261.70) | 218.57 (164.71, 253.68) | 184.54 (136.17, 293.77) | <.001 |
| LHR | 1.87 (1.45, 2.60) | 2.01 (1.68, 2.50) | 1.56 (1.30, 2.60) | <.001 |
| TYG | 1.92 (1.48, 2.62) | 2.05 (1.54, 2.58) | 1.81 (1.31, 2.68) | 0.002 |
| CHG | 2.96 (2.68, 3.32) | 2.95 (2.68, 3.27) | 3.04 (2.70, 3.39) | 0.801 |
| WHTR | 0.62(0.59, 0.71) | 0.60(0.53, 0.66) | 0.67(0.63, 0.73) | <.001 |
| METS-IR | 2.27(2.36, 2.62) | 2.28(2.38, 2.42) | 2.31(2.39, 2.59) | 0.018 |
| METS-VF | 7.01(6.47, 7.24) | 6.82(6.48, 7.03) | 7.11(6.44, 7.31) | <.001 |

Notes: Data are shown as median with interquartile range (IQR) for continuous variables and number with percentage for categorical variables. Abbreviations: BMI, body mass index; SFA-L3, subcutaneous fat area of the third lumbar vertebra; VFA-L3, visceral fat area of the third lumbar vertebra; CSA-L3 cross sectional area of the third lumbar vertebra; SMI-L3, skeletal muscle index of the third lumbar vertebra; RBC, red blood cell count; HGB, hemoglobin; PLT, platelet count; NEUT, neutrophil count; PLT, platelet count; LYMPH, lymphocyte count; MONO, monocyte count; FBG, fasting plasma glucose; PBG, postprandial blood glucose; HbA1c, haemoglobin A1c; FCP, fasting c peptide; FINS, fasting insulin; TG, triglycerides; TC, total cholesterol; Cr, creatinine; UA, uric acid; HDLC, high-density lipoprotein cholesterol; LDL-C, low-density lipoprotein cholesterol; ALT, alanine aminotransferase; ALP, alkaline phosphatase; AST, aspartate aminotransferase; GGT, Gamma glutamyl transferase; LDH, lactate dehydrogenase; Na, natrium; Ca, calcium; Pi, phosphorus; Mg, magnesium; AFP alpha-fetoprotein; CA125, carbohydrate antigen 125; CA199, Carbohydrate Antigen 19-9; CEA, MHR, Monocyte-to-HDL Ratio; NHR, Neutrophil-to-HDL Ratio; PHR, Platelet-to-HDL Ratio; LHR, Lymphocyte-to-HDL Ratio; TyG, Triglyceride-Glucose Index; CHG, Cholesterol-Glucose Index; WHTR, waist-to-height ratio;METS-IR, metabolic score for insulin resistance; METS-VF, metabolic score for visceral fat;

**Table S2 LASSO selection of variables and covariance analysis.**

| Variables | Coefficient of LASSO | VIF |
| --- | --- | --- |
| Age* | -0.327 | 1.653 |
| CSA-L3 | 0.0 | 3.736 |
| RBC | 0.0 | 1.856 |
| HGB* | -0.015 | 1.359 |
| ALT | 0.0 | 1.249 |
| BMI | 0.0 | 1.133 |
| SMI-L3* | -0.05 | 3.310 |
| METS-VF* | -0.45 | 1.317 |
| HbA1c | 0.0 | 1.246 |
| NEUT* | 0.265 | 1.025 |
| TG | 0.0 | 1.656 |
| TC | 0.0 | 1.240 |
| UA* | -0.937 | 1.123 |
| CEA | 0.067 | 1.545 |
| Gender | 0.02 | 2.331 |
| AST | 0.0 | 1.476 |
| ALP* | -0.03 | 1.558 |
| TyG | 0.0 | 1.479 |
| CHG | 0.0 | 1.989 |
| LHR* | -0.034 | 1.103 |

* Feature variables further identified by stepwise logistic regression.

VIF < 5 is considered to be non-multicollinear.

Abbreviations: LASSO, least absolute shrinkage and selection operator; VIF, variance inflation factor; BMI, body mass index; CSA-L3 cross sectional area of the third lumbar vertebra; SMI-L3, skeletal muscle index of the third lumbar vertebra; RBC, red blood cell count; HGB, hemoglobin; NEUT, neutrophil count; HbA1c, haemoglobin A1c; TG, triglycerides; TC, total cholesterol; UA, uric acid; ALT, alanine aminotransferase; ALP, alkaline phosphatase; AST, aspartate aminotransferase; CEA, carcinoembryonic antigen; Lymphocyte-to-HDL Ratio; TyG, Triglyceride-Glucose Index; CHG, Cholesterol-Glucose Index; METS-VF, metabolic score for visceral fat;

**Table S3 Tuning ranges and optimal hyperparameters for each model.**

| Model | Hyperparameter | Tuning range | Optimal value |
| --- | --- | --- | --- |
| XGB | n estimators | - | 20 |
|  | learning rate | seq(0.01,0.4,by=0.01) | 0.1 |
|  | max depth | seq(2,10,by=1) | 4 |
|  | min child weight | c(1,2,3,4,5,6) | 6 |
|  | gamma | c(seq(0,1,by=0.05),3,5) | 0.5 |
|  | subsample | seq(0.05,1,by=0.05) | 1 |
|  | colsample bytree | seq(0.3,1.0,by=0.1) | 1 |
|  | reg lambda | seq(0,10,by=0.5) | 0.5 |
|  | booster | - | gbtree |
| LR | C | - | 0.09999999999999999 |
|  | l1_ratio | - | None |
|  | max_iter | - | 50 |
|  | penalty | - | l2 |
|  | solver | - | lbfgs |
|  | tol | - | 0.0001 |
| SVM | kernal | - | rbf |
|  | C | 2^seq(log2(0.1),log2(40),by=0.5) | 1.0 |
|  | gamma | 2^seq(-10,-5,by=0.5) | scale |
|  | probability | - | True |
| GBDT | n_estimators | - | 1 |
|  | loss | - | log_loss |
|  | max depth | c(4,8,16) | 1 |
|  | min_samples_leaf | c(0.5,1.0,2.0,3.0) | 1 |
|  | min_samples_split | c(0.5,1.0,2.0,3.0) | 2 |
|  | learning rate | seq(0.05,2,by=0.01) | 2 |
| MLP | hiden layzer sizes | list(c(10),c(20),c(30),c(60),c(16,16),c(24,12),c(32,16),c(32,32),c(64,64),c(128,128)) | 30 |
|  | activation | Rectifier,Tanh,TanhWithDropout,Maxout | tanh |
|  | learning rate | c(0.001,0.005,0.01,0.05,0.1) | constant |
|  | epoch | c(5,10,15,20,30,40,50) | 20 |
|  | input dropout ratio | c(0.01,0.02,0.1,0.2) | 0.01 |
| GNB | laplace | c(0,1,2,3) | 1 |
|  | var_smoothing | - | 1e-07 |
|  | adjust | seq(1,4,by=0.1) | 2 |
| LightGBM | num leaves | c(2,3,4) | 4 |
|  | max depth | c(4,8,16) | 1 |
|  | min data in leaf | c(15,20,25,30,35,40) | 25 |
|  | learning rate | seq(0.05,2,by=0.01) | 2 |
|  | n estmators | - | 5 |
|  | lambda l1 | c(0,0.1,0.5,1) | 1 |
|  | lambda l2 | c(0,0.1,0.5,1) | 1 |
|  | feature fraction | c(0.4,0.5,0.6,0.7,0.8,0.9) | 0.9 |
|  | bagging fraction | c(0.6,0.8,0.9) | 0.9 |
|  | boosting_type | - | gbdt |


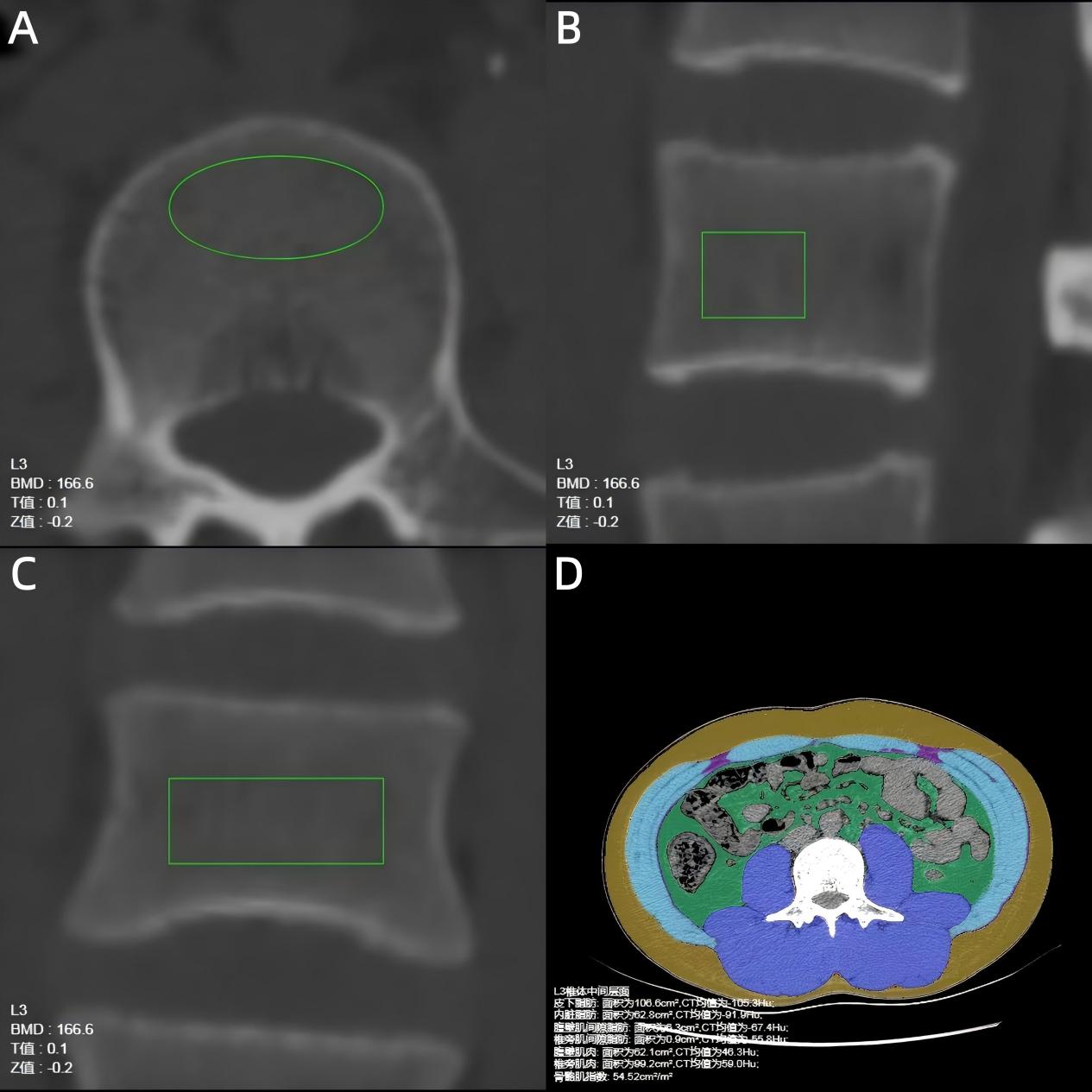


Figure S1. Automatic segmentation of abdominal tissues at the L3 vertebral level on CT. A. Axial view at the L3 vertebral level; B. Sagittal view at the L3 vertebral level; C. Coronal view at the L3 vertebral level; D. Automatic segmentation of abdominal tissues performed on the axial image at the L3 vertebral level.


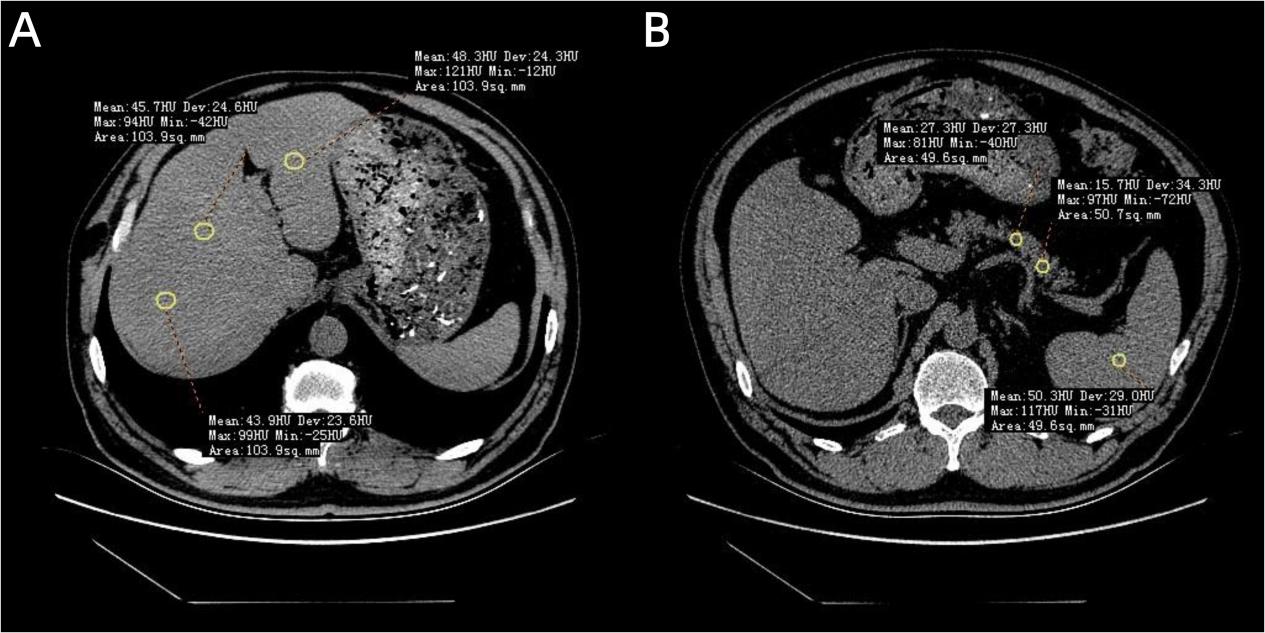


Figure S2. Schematic diagram of CT attenuation measurement in the liver (A), pancreas and spleen (B).


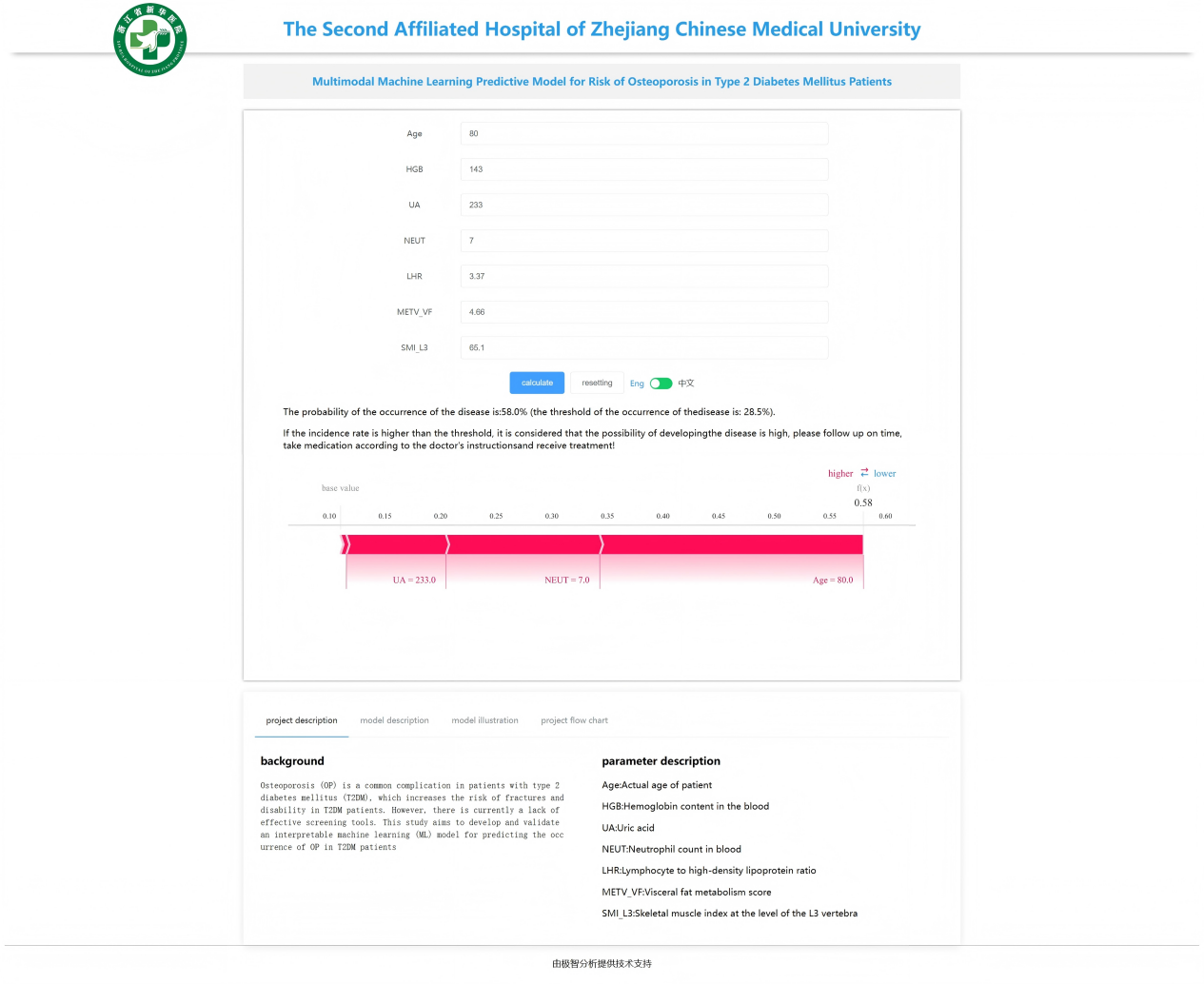


Figure S3. Interface of the Multimodal Machine Learning Predictive Model for Osteoporosis Risk in Middle-aged and Elderly Patients with Type 2 Diabetes Mellitus.
